# Effects of trait stacking on the maize phyllosphere microbiome: a shotgun metagenomic comparison

**DOI:** 10.3389/fmicb.2026.1904641

**Published:** 2026-09-28

**Authors:** Jing Ying Yan, Yuan Zhou, Liang Ni, Zhong Jie Ding, Chaoyang Lin

**Affiliations:** 1 Agricultural Experiment Station, Zhejiang University, Hangzhou, China; 2 Comprehensive Technical Service Center of Zhoushan Customs, Zhoushan, China; 3 State Key Laboratory of Plant Environmental Resilience, College of Life Sciences, Zhejiang University, Hangzhou, China; 4 Institute of Agricultural and Rural Development, Zhejiang University, Hangzhou, China

**Keywords:** biosafety assessment, metagenomics, microbiome, phyllosphere, transgene

## Abstract

Current safety assessments for stacked-trait transgenic crops underrepresent the phyllosphere, a critical interface directly exposed to transgenic products, warranting strengthened risk evaluation. Here, we employed metagenomics to characterize the phyllosphere of three maize lines under herbicide-free conditions: wild-type (Corn.CK), herbicide-resistant single-trait (transgene of *cp4-epsps*, Corn.S), and insect- and herbicide-resistant stacked-trait (transgene of *cry1Ab* and *cp4-epsps*, Corn.D). Shannon diversity showed a consistent trend toward higher values in the *cp4-epsps* single-trait line than in the wild type (not significant after correction for multiple comparisons), whereas the stacked-trait line showed an intermediate increase. Transgenic events were associated with bidirectional taxonomic remodeling, characterized by the depletion of Enterobacterales and enrichment of environmentally adapted taxa. Functionally, transgenic lines displayed pervasive metabolic depletion across KEGG pathways and CAZyme categories, alongside reduced abundance of virulence-associated gene homologs (e.g., T6SS and Enterobactin) and lower antibiotic resistance gene diversity (wild-type > stacked-trait > single-trait), driven by the concomitant loss of Enterobacterales hosts. Notably, in most of the taxonomic, functional, virulence, and resistome dimensions examined, the stacked-trait line exhibited an intermediate microbiome profile between the wild-type and single-trait lines. These findings suggest that, for this genotype combination under these controlled conditions, trait stacking was not associated with microbiome perturbation greater than that observed for the single trait, and provide a preliminary, genotype- and stage-specific reference for phyllosphere biosafety assessment, pending field validation.

## Introduction

Plants form intimate symbiotic relationships with microbial communities, and the plant microbiome plays important roles in nutrient acquisition, growth regulation, and stress defense (Vandenkoornhuyse et al., 2015; Fitzpatrick et al., 2020). Metagenomics technology has overcome the limitations of species-level taxonomy, enabling direct reconstruction of gene functional profiles and revealing microbial mechanisms in metabolic pathways, resistance, and environmental adaptation (Vorholt, 2012). Among plant microbiome studies, the rhizosphere and phyllosphere represent fundamentally distinct ecological niches. Rhizosphere communities are driven by soil buffering and root exudates, and their assembly is governed largely by deterministic processes. The phyllosphere, by contrast, is a distinct microbial habitat whose community assembly is jointly driven by stochastic dispersal and environmental filtering (Vorholt, 2012).

The phyllosphere microbial community is dominated by Pseudomonadota (formerly Proteobacteria), especially Methylobacterium, Sphingomonas, and Pseudomonas, followed by Bacillota (formerly Firmicutes) and Actinomycetota (formerly Actinobacteria), with fungal communities predominantly composed of Ascomycota. Metagenomic analyses have revealed metabolic cross-feeding relationships among core taxa, such as carbon source exchange between methylotrophs and Pseudomonas (Delmotte et al., 2009). Beyond core taxa, the phyllosphere harbors rare species, protists, and bacteriophages that may contribute to community functional compensation (Flues et al., 2018; Kwon et al., 2018). The phyllosphere also exhibits spatial heterogeneity, with differentiation between epiphytic and endophytic compartments in both taxonomic composition and functional potential (Vionnet et al., 2018; Zhu et al., 2018; Kirschner et al., 2019). Additionally, phyllosphere microbial diversity shows temporal dynamics: community assembly in nascent leaves is largely stochastic, whereas with increasing leaf age it becomes governed by environmental filtering, with seasonal fluctuations as core drivers (Laforest-Lapointe et al., 2016). Notably, the high taxonomic diversity of the phyllosphere often masks functional redundancy, where different taxonomic groups may perform similar metabolic functions (Louca et al., 2018). This coexistence of high spatiotemporal variation and functional redundancy implies that weak genotypic signals (such as transgenic events) can be overwhelmed by environmental “noise” (Liu et al., 2020), making high signal-to-noise controlled environments essential for detecting such effects.

In the heterogeneous phyllosphere, microorganisms have evolved adaptive strategies. Dominant groups such as Sphingomonas exhibit stress response signatures at the proteome level, including carotenoid synthesis for antioxidant defense and DNA repair mechanisms (Delmotte et al., 2009). Sphingomonas also competes for iron by secreting siderophores and produces antimicrobial substances to inhibit pathogen colonization (Innerebner et al., 2011). Phyllosphere microorganisms migrate directionally via chemotaxis, attach irreversibly using pili, and secrete extracellular polysaccharides to form biofilms (Hirano and Upper, 2000), while accumulating compatible solutes to cope with drought stress (Genitsaris et al., 2020). These functional traits enable phyllosphere microorganisms to contribute to maintaining ecosystem health and plant disease resistance (Rosado et al., 2018; Sohrabi et al., 2023). However, the above understanding is largely derived from model plants or natural ecosystems. In agricultural systems, particularly regarding transgenic crops, such in-depth functional characterization of the phyllosphere is lacking.

The global cultivation area of transgenic crops has continued to increase, with 28 countries achieving commercial cultivation in 2024, covering 209.8 million hectares (Li et al., 2024). The plant microbiome plays a role in community assembly and plant health maintenance (Trivedi et al., 2020). However, the existing microecological safety assessment system exhibits a notable ecological niche bias: most research has focused on rhizosphere microbial responses (Widmer, 2007), while systematic studies of the phyllosphere, the direct site of transgenic product expression, remain scarce. Rhizosphere microorganisms are buffered by soil, such that transgenic effects are often attenuated (Widmer, 2007). Consequently, rhizosphere conclusions cannot be simply extrapolated to the phyllosphere, which lacks soil buffering and constitutes a transient, oligotrophic microenvironment directly regulated by the host (Vorholt, 2012). More critically, stacked-trait crops have become the breeding mainstream. Although gene pyramiding effectively delays pest resistance (Tabashnik et al., 2013), multiple genetic modifications may impose superimposed pressures on host metabolic networks, producing synergistic or antagonistic effects on phyllosphere microorganisms (Icoz and Stotzky, 2008). The few existing stacked-trait studies mostly employ a “stacked trait vs. wild-type” binary comparison, lacking single-gene controls, making it impossible to distinguish the marginal contribution differences between single and stacked traits. Furthermore, for herbicide-resistant crops, microbial community fluctuations observed in the field often originate from secondary effects of glyphosate application rather than the *cp4-epsps* gene *per se* (Aristilde et al., 2017). Without isolating this confounding variable, the net effect of the transgene on phyllosphere microbial communities cannot be clarified.

The application of metagenomics in transgenic safety assessment also exhibits methodological lag. Phyllosphere research remains predominantly reliant on 16S rRNA amplicon sequencing, while metagenomic studies are primarily concentrated in the rhizosphere (Widmer, 2007; Redford et al., 2010). Three challenges are central. Phyllosphere samples have low microbial biomass and suffer severe host DNA interference (Aristilde et al., 2017). Spatiotemporal heterogeneity is also high, with environmental factors explaining far more community structure than host genotype (Eisenhofer et al., 2019), which risks “false negative” conclusions (Marvier, 2002). Finally, the confounding effects of agronomic management are difficult to isolate (Redford et al., 2010). Even in rhizosphere studies, existing assessments often lack single-gene controls, making it impossible to distinguish marginal contribution differences between single and stacked traits (Andow and Zwahlen, 2006; Yang et al., 2023). Recent advances in host DNA removal technologies and deep sequencing strategies are gradually overcoming these methodological bottlenecks.

Addressing these challenges, this study used a controlled greenhouse environment to reduce environmental interference and establish a descriptive baseline of genotype-associated differences in the phyllosphere microbiome. This follows the recommendation that controlled-environment experiments are a necessary prerequisite for causal inference in phyllosphere microbiome research (Laforest-Lapointe et al., 2016). Maize near-isogenic lines (NILs) were utilized to construct a gene quantity gradient comprising three genotypes: wild-type (non-transgene, Corn.CK), herbicide-resistant single trait (transgene of *cp4-epsps*, Corn.S), insect-resistant and herbicide-resistant stacked trait (transgenes of *cry1Ab* and *cp4-epsps*, Corn.D). Within this framework, the central objective was to characterize the microbiome profile of the stacked-trait line relative to the wild-type and single-trait lines. Operationally, we defined an intermediate phenotype as a pattern in which Corn.D lies between Corn.CK and Corn.S, meaning that the stacked-trait line falls closer to the wild-type baseline than the single-trait line does. This pattern was assessed consistently across independent analytical dimensions, namely taxonomic composition, functional potential, virulence factors, and the antibiotic resistome. Through herbicide-free greenhouse management, agronomic interference was thereby minimized, enabling a controlled descriptive comparison among the three genotypes. This characterization establishes a controlled baseline against which the phyllosphere biosafety of stacked-trait transgenic crops can be evaluated.

## Materials and methods

### Plant materials and greenhouse cultivation

Maize near-isogenic lines were used in this study to exclude genetic background noise. The three lines were derived from a single backcrossing pedigree. Briefly, a commercial stacked-trait hybrid (NK603 × MON810; NK603 carries the *cp4-epsps* event, and MON810 carries the *cry1Ab* event) was crossed with the maize variety Shen 3,336, which served as the recurrent parent. The F₁ progeny, which carried both transgenes in the heterozygous state on the Shen 3,336 background, were repeatedly backcrossed to Shen 3,336 for seven generations (BC1–BC7). In each backcross generation, progeny carrying the transgene(s) of interest were identified by PCR-based genotyping and used as pollen donors for the next backcross. The BC7 plants were self-pollinated to fix the genotypes, and three genotypic classes were separated from the BC7 selfed progeny: Corn.CK (no transgene), Corn.S (*cp4-epsps* only), and Corn.D (*cry1Ab* + *cp4-epsps*). All three lines share a largely common Shen 3,336 genetic background: apart from the transgene region(s), they are expected to share >99% genome identity, with any residual donor genome expected to be concentrated around the transgene insertion sites (linkage drag). Because the genome-wide extent of the introgressed donor segments was not mapped, the genotype-associated differences reported here may in principle include contributions from linked donor segments in addition to the transgene(s) themselves. All seeds were provided by the same breeding institution and surface-sterilized (2.5% NaClO for 5 min, 75% ethanol for 2 min, followed by five sterile water rinses) before sowing in a sterilized substrate.

To minimize field environmental noise and the confounding effects of agronomic management (e.g., herbicide application), plants were cultivated in a controlled greenhouse. Conditions were set as follows: photoperiod of 16 h light/8 h dark, temperature of 28 °C (day)/22 °C (night), relative humidity of 60%, and light intensity of 800 μmol·m^−2^·s^−1^. No herbicides, insecticides, or fungicides were applied throughout the growth period; only sterile deionized water was supplied. Three biological replicates were established for each genotype in a completely randomized design: the nine experimental units were assigned to greenhouse positions at random, with no blocking factor applied, and genotype was accordingly the only factor included in the subsequent statistical analyses.

### Phyllosphere sample collection

Sampling was conducted between 9:00 and 11:00 a.m. during the V7 vegetative stage. Each biological replicate consisted of leaf material pooled from five randomly selected plants of the same genotype: from each plant, the penultimate fully expanded leaf was excised at its middle 15-cm segment using sterile surgical scissors, and the five segments were combined into a single sterile 50 mL tube to form one biological replicate. The three biological replicates per genotype were formed from non-overlapping sets of randomly selected plants (15 individual plants per genotype; 45 plants in total), such that each pooled sample was independent of the others. Pooling was intended to integrate plant-to-plant variability within each genotype and to yield a representative community profile per replicate. Operators wore sterile protective clothing and nitrile gloves, changing gloves between plants. Pooled samples were immediately flash-frozen in liquid nitrogen. Microbial extraction and metagenomic sample preparation were completed by Beijing Novogene Corporation.

### Metagenomic sequencing and bioinformatic analysis

PE150 paired-end sequencing was performed on the Illumina NovaSeq 6,000 platform. Each sample was sequenced to a depth of no less than 6 Gb to ensure coverage of low-abundance functional genes. Given the low-biomass nature of phyllosphere samples, a host DNA depletion strategy was employed: the NEBNext Microbiome DNA Enrichment kit was used to enrich microbial DNA and reduce host genomic background interference. The raw data quality control and bioinformatic analysis pipeline proceeded as follows: starting from raw data, fastp v0.23.2 was used for quality filtering (Q20 threshold) and adapter removal, yielding clean data. Bowtie2 v2.4.5 was used to map clean data against the maize reference genome (B73 RefGen_v4) to remove host-derived reads. The filtered data were co-assembled using MEGAHIT v1.2.9, followed by gene prediction using MetaGeneMark v3.38, with dereplication at 95% clustering similarity to obtain the unigene catalog (non-redundant gene set).

Based on the unigene catalog, gene sequences were aligned to the NCBI NR database using Diamond v2.0.15 for taxonomic annotation (*e*-value threshold 1e-5, best hit selected), and species classification was performed using the LCA algorithm in MEGAN6. Multiple databases were utilized for functional analysis, including KEGG (metabolic pathways, v96.0), VFDB (virulence factor database, 2022 update), eggNOG (functional classification, v5.0), PHI (pathogen-host interaction database, v4.14), CAZy (carbohydrate-active enzyme database), CARD (comprehensive antibiotic resistance database, v3.2.5), and MGEs (mobile genetic element database). All functional annotations were filtered using thresholds of e-value < 1e-5, identity > 30%, and coverage > 50%. Because species-level pathogen assignment is more sensitive to sequence similarity than functional annotation, a stricter identity threshold of > 50% was additionally applied to the analysis of pathogenicity-associated genes using the PHI database. This removed species-level assignments to taxa with no plausible ecological niche in the phyllosphere, while leaving the pathogen profiles otherwise essentially unchanged.

Annotation results were further subjected to three categories of in-depth analyses: (1) Species and functional diversity analysis, including Alpha diversity (Shannon index, Simpson index, Chao1 index; compared among genotypes using the Kruskal–Wallis rank-sum test, with individual pairwise contrasts evaluated by two-sided exact two-sample permutation tests and Holm–Bonferroni correction applied across the three pairwise comparisons within each diversity index), PCA, cluster analysis, PCoA (based on Bray-Curtis distance of genus-level relative abundance profiles), and NMDS (based on Bray-Curtis distance, using the metaMDS function with 500 iterations). The robustness of the Kruskal–Wallis results was additionally confirmed by exact permutation tests, based on exhaustive enumeration of all 1,680 possible assignments of the nine samples to the three genotype groups. Each pairwise contrast (Corn.S vs. Corn.CK, Corn.D vs. Corn.CK, Corn.D vs. Corn.S) was then evaluated with a two-sided exact two-sample permutation test (Fisher–Pitman permutation test on the difference in group means), in which the exact null distribution was obtained by exhaustive enumeration of all C(6,3) = 20 possible allocations of the six samples of each pair; this analysis is equivalent to the exact Fisher–Pitman test implemented by the R package coin (oneway_test, distribution = “exact”). An exact rank-based permutation test (Wilcoxon rank-sum on mid-ranks, with ties handled by the exact permutation distribution) was computed as a confirmatory sensitivity analysis and yielded identical conclusions. Benjamini–Hochberg FDR adjustment was additionally computed as a complementary sensitivity analysis and yielded the same conclusions. Because only n = 3 biological replicates per genotype were available, the smallest attainable two-sided exact permutation *p*-value is 2/20 = 0.100 (Holm-adjusted minimum, 0.300); the pairwise tests, therefore, quantify the strength of evidence for individual contrasts rather than declare significant differences. (2) Differential analysis between groups, using ANOSIM (999 permutations) and LEfSe (LDA threshold > 2, *p* < 0.05). (3) Antibiotic resistance gene analysis, encompassing functional diversity analysis of resistance genes, inter-group differential analysis, and association analysis between resistance genes and species, and between resistance genes and mobile genetic elements (based on Spearman correlation, |*r*| > 0.6 and FDR < 0.05). Mobile genetic elements were additionally profiled by aligning the unigene catalog against the INTEGRALL (integrons), ISfinder (insertion sequences), and plasmid databases, and abundances were summed within each MGE class per sample. ARG–MGE co-occurrence was then evaluated by Spearman correlation across the nine samples, both between total ARG and total MGE abundance and for each ARG against each MGE class, with Benjamini–Hochberg FDR correction and co-occurrence defined as |*r*| ≥ 0.6 and FDR < 0.05. Taxonomic annotation resolution was quantified from the MEGAN LCA output as the proportion of the total gene abundance classified at each rank, expressed both relative to the entire non-redundant gene catalog and relative to the taxonomically assigned genes. All statistical analyses were performed in the R v4.2.1 environment using packages including vegan, phyloseq, coin, and ggplot2 for visualization.

## Results

### Alpha and beta diversity of the phyllosphere microbiome

Metagenomic sequencing yielded high-quality data across all samples (>Q30; Supplementary Figure S1A), and the assembled non-redundant gene catalog is summarized in Supplementary Figures S1B, C. Across the non-redundant gene catalog, 25.3% of the gene abundance received a taxonomic assignment (to at least the domain level), consistent with the low-biomass nature of phyllosphere samples and the incomplete representation of leaf-associated taxa in reference databases; among taxonomically assigned genes, 89.5, 76.8 and 59.0% were resolvable to the phylum, genus and species levels, respectively. The overall assignment rate declined from the wild type to the transgenic lines (Corn.CK 33.4% > Corn.D 24.0% > Corn.S 18.6%), mirroring the depletion of abundant, well-characterized Enterobacterales, whereas the proportion of the catalog resolved to the species level was comparable across genotypes (approximately 15%; Supplementary Figure S2). Alpha diversity analysis revealed differences across the three maize lines (Figure 1). The Shannon diversity index followed the gradient Corn.S > Corn.D > Corn.CK (Figure 1A; Kruskal–Wallis omnibus *p* = 0.079). In exact two-sample permutation tests, however, no individual pairwise contrast reached statistical significance: the increase over Corn.CK was not significant for either Corn.S (exact permutation *p* = 0.100; Holm-adjusted *p* = 0.300) or Corn.D (*p* = 0.200; Holm-adjusted *p* = 0.400), and Corn.S and Corn.D did not differ significantly (*p* = 0.300; Holm-adjusted *p* = 0.400). The Simpson diversity index showed a similar but non-significant trend (Figure 1B; Kruskal–Wallis *p* = 0.491; exact permutation *p* = 0.543; exact pairwise permutation *p* = 0.300–0.600, Holm-adjusted *p* = 0.900 for all three contrasts). Effect sizes were consistent across both evenness indices: relative to Corn.CK, the shift was largest in Corn.S (Shannon, Cliff’s δ = +1.00; Simpson, δ = +0.56) and smaller in Corn.D (δ = +0.78 and +0.33, respectively), with Corn.D falling between the two other lines (versus Corn.S, δ = −0.56 and −0.33). These patterns indicate that the increase in community evenness relative to the wild type was most pronounced for the *cp4-epsps* single-trait line, whereas trait stacking conferred no additional increase. Because only *n* = 3 biological replicates per genotype were available, the smallest attainable two-sided exact permutation *p*-value is 2/20 = 0.100 (Holm-adjusted minimum, 0.300), and the alpha-diversity patterns are therefore interpreted as consistent descriptive trends supported by large effect sizes rather than statistically confirmed differences. Additionally, the Chao1 richness estimator followed a different pattern (Supplementary Figure S3A; Kruskal–Wallis *p* = 0.358; exact permutation *p* = 0.457): Corn.D exhibited the highest richness, showing higher values than both Corn.CK (Cliff’s δ = +0.43; exact permutation *p* = 0.700; Holm-adjusted *p* = 1.000) and Corn.S (δ = +1.00; *p* = 0.300; Holm-adjusted *p* = 0.900), whereas richness in Corn.CK and Corn.S was comparable (δ = −0.14, negligible; *p* = 1.000; Holm-adjusted *p* = 1.000). In contrast to the evenness indices, the stacked-trait line was not intermediate for richness. This decoupling of evenness and richness underscores the importance of employing multiple diversity metrics, as single indices may mask ecologically meaningful patterns.

**Figure 1:**
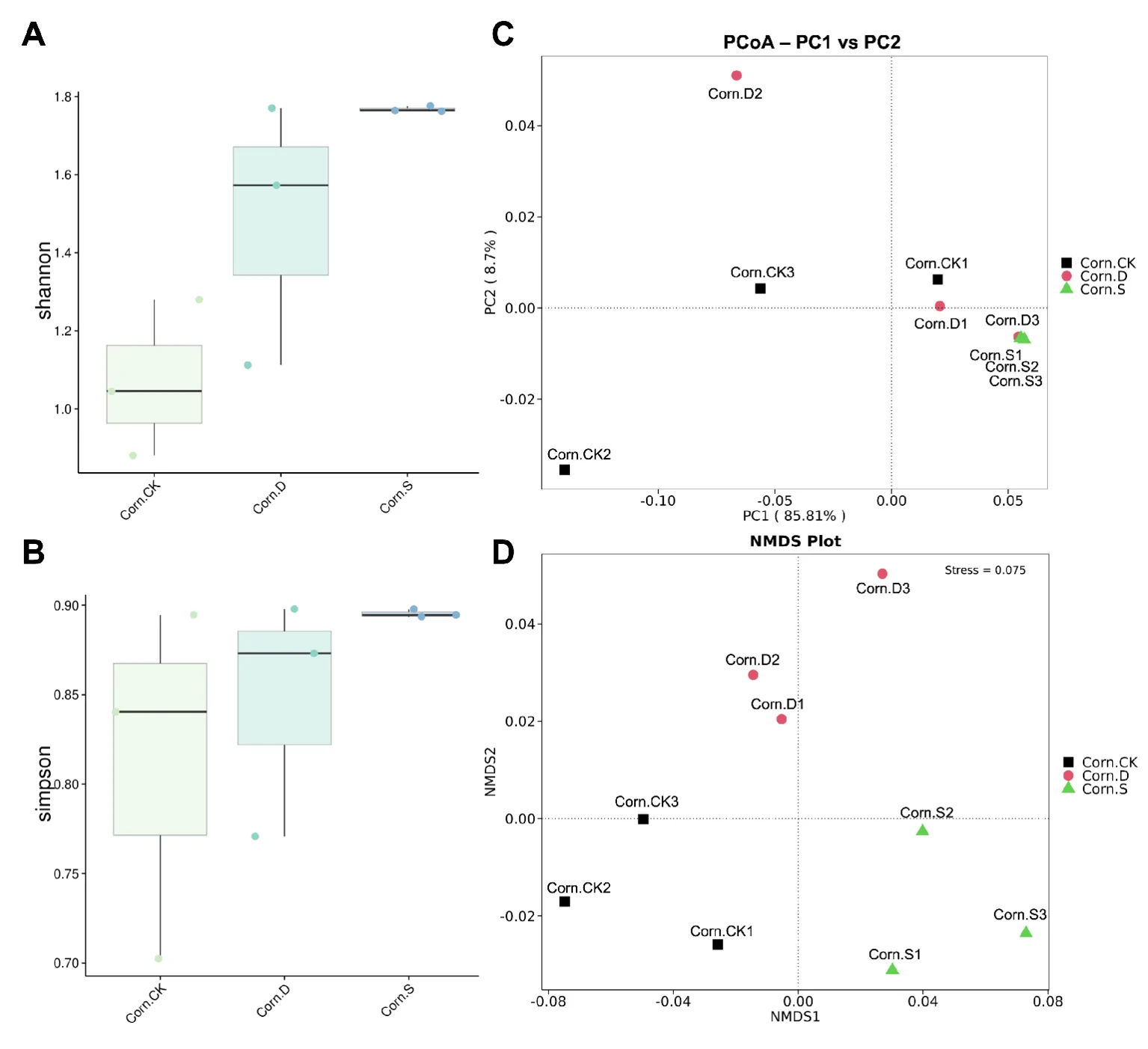
Microbial diversity analysis of the phyllosphere microbiome across transgenic maize lines. **(A)** Shannon diversity index comparison among Corn.CK, Corn.S, and Corn.D groups. Cliff’s δ: Corn.S versus Corn.CK, +1.00; Corn.D versus Corn.CK, +0.78; Corn.D versus Corn.S, −0.56. **(B)** Simpson diversity index comparison among the three groups. Cliff’s δ: Corn.S versus Corn.CK, +0.56; Corn.D versus Corn.CK, +0.33; Corn.D versus Corn.S, −0.33. **(C)** Principal Coordinates Analysis (PCoA) based on Bray–Curtis dissimilarity of genus-level relative abundance profiles, showing community-level dissimilarities among individual samples. **(D)** Non-metric Multidimensional scaling (NMDS) analysis based on Bray-Curtis distance (stress = 0.075) illustrating community structure differences among groups. Alpha diversity indices in **(A, B)** were compared among the three groups using the Kruskal–Wallis rank-sum test, the robustness of which was confirmed by exact permutation tests enumerating all 1,680 possible assignments of the nine samples to the three genotype groups (Shannon, *p* = 0.079; exact permutation *p* = 0.086; Simpson, *p* = 0.491; 0.543). Each pairwise contrast (Corn.S vs. Corn.CK, Corn.D vs. Corn.CK, Corn.D vs. Corn.S) was then tested with a two-sided exact two-sample permutation test based on exhaustive enumeration of all 20 possible allocations of the six samples of each contrast, with Holm–Bonferroni correction applied across the three pairwise contrasts per index. All pairwise comparisons were non-significant (exact permutation *p* ≥ 0.100; Holm-adjusted *p* ≥ 0.300; for the Shannon index, Corn.S versus Corn.CK: exact permutation *p* = 0.100, Holm-adjusted *p* = 0.300). With *n* = 3 biological replicates per group, the smallest attainable two-sided exact permutation *p*-value is 2/20 = 0.100. Panels **(C, D)** are ordination analyses presented descriptively and are not associated with a significance test. *n* = 3 biological replicates per group throughout. Each replicate consists of pooled penultimate-leaf segments from five randomly selected plants. Effect sizes are Cliff’s δ (Cliff, 1993), with positive values indicating higher values in the first-named group and tied pairs excluded (|δ| < 0.147 negligible, < 0.33 small, < 0.474 medium, ≥ 0.474 large); at *n* = 3, δ = 1.00 corresponds to an exact two-sided *p*-value of 0.10.

Beta diversity analysis was performed to explore patterns of community structure among the three groups. PCoA of genus-level community profiles illustrated a pattern of separation, with PC1 (85.81% variance) positioning Corn.CK apart from the transgenic lines and PC2 (8.70%) partially separating Corn.D and Corn.S (Figure 1C). The uneven split of variance between the two axes is itself informative. The dominant axis of variation in the phyllosphere community is the presence or absence of transgenes, whereas the distinction between the single- and stacked-trait lines, though resolved along PC2, accounts for roughly an order of magnitude less of the total variation. NMDS analysis showed a visually consistent pattern (stress = 0.075; Figure 1D), with Corn.CK clustering distinctly from both transgenic lines, and Corn.D and Corn.S showing moderate separation along NMDS2. ANOSIM analysis showed a moderate separation trend between Corn.CK and Corn.S (*R* = 0.556, *p* = 0.1) but no separation between Corn.CK and Corn.D (*R* = 0, *p* = 0.3; Supplementary Figures S3B, C), likely reflecting limited sample size and high within-group variability. Notably, although neither comparison reached statistical significance, the ANOSIM R statistic itself, which reflects effect size independently of significance, differed considerably between the two (*R* = 0.556 versus *R* = 0), suggesting a greater degree of community separation between Corn.CK and Corn.S than between Corn.CK and Corn.D. Collectively, ordination analyses illustrate patterns of community separation that appear to align primarily with the wild-type–transgenic distinction, although these are descriptive visualizations rather than statistically confirmed differences. The lack of significant differentiation between Corn.D and Corn.S, alongside the intermediate position of Corn.D in ordination analyses, is descriptively consistent with an intermediate community structure of the stacked-trait line relative to the wild-type and single-trait lines, though this observation is not statistically confirmed.

### Bidirectional taxonomic remodeling in the transgenic phyllosphere

Phylum-level analysis revealed detectable taxonomic shifts across the three maize lines (Figure 2A). Pseudomonadota was the dominant bacterial phylum, with higher relative abundance in Corn.CK than in both transgenic lines. Other phyla (Bacteroidota, Bacillota, Actinomycetota, Basidiomycota, and Mucoromycota) remained relatively consistent across groups. Notably, Corn.D exhibited an intermediate phylum-level composition, with Pseudomonadota abundance lower than Corn.CK but higher than Corn.S. Class-level analysis revealed that the Pseudomonadota depletion was driven by reduced Gammaproteobacteria in transgenic lines (Supplementary Figure S4A). Order-level analysis further identified Enterobacterales as the primary lineage depleted in transgenic lines, rather than a broad reduction across all proteobacterial orders (Supplementary Figure S4B).

**Figure 2:**
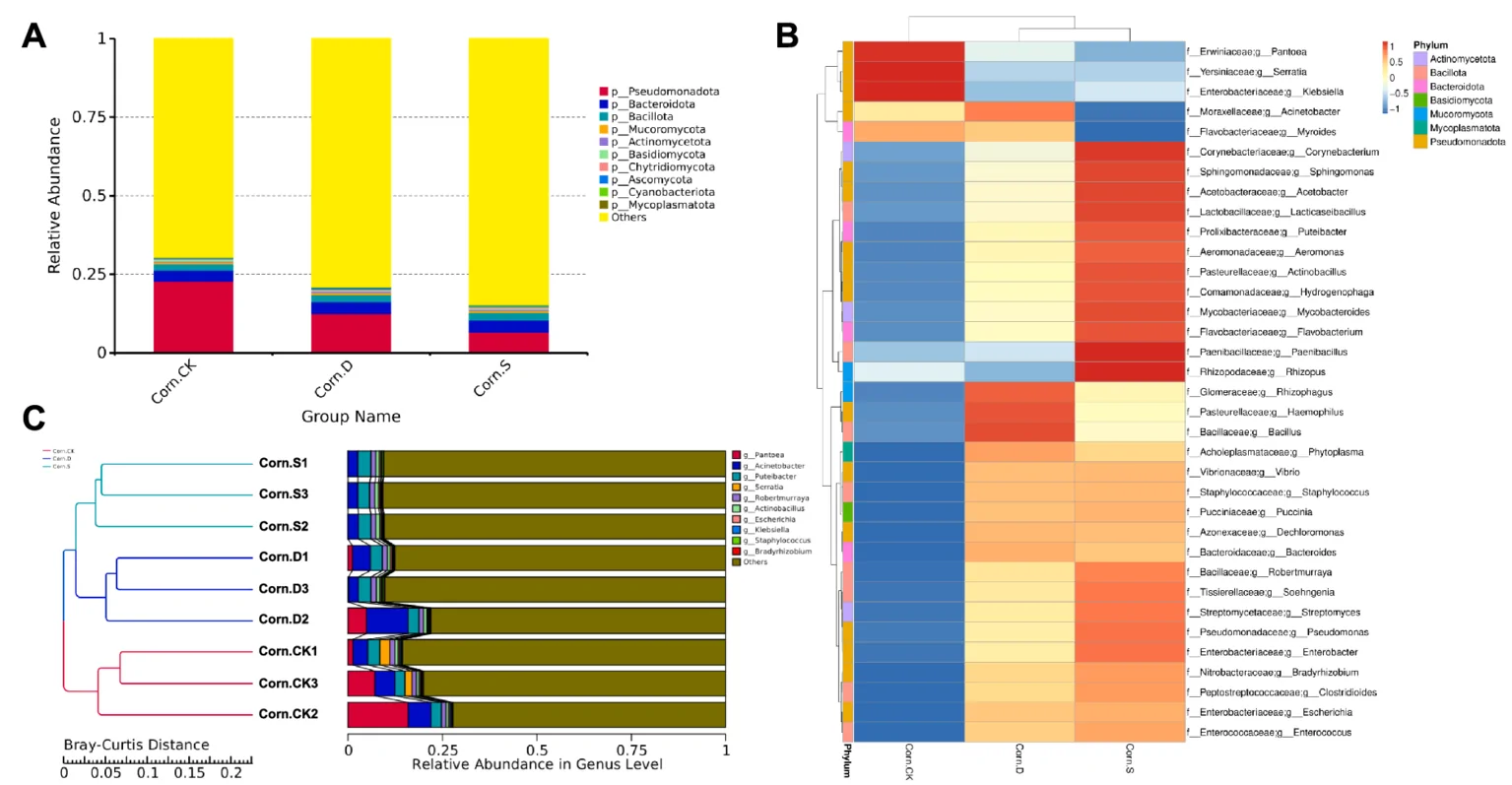
Taxonomic composition of the phyllosphere microbiome across transgenic maize lines. **(A)** Phylum-level relative abundance (stacked bar chart) showing the taxonomic distribution across Corn.CK, Corn.S, and Corn.D. **(B)** Genus-level heatmap with hierarchical clustering revealing abundance patterns across groups. **(C)** Cluster analysis (based on Bray-Curtis distance) and stacked bar plot of relative abundance at the genus level, illustrating the similarity of microbial community composition among different groups. Panels **(A–C**) are descriptive summaries of relative abundance and are not associated with significance testing. n = 3 biological replicates per group.

Genus-level heatmap analysis revealed pronounced differentiation between the wild-type and transgenic phyllosphere (Figure 2B). Enterobacterales-associated genera, including Pantoea and Serratia, were enriched in Corn.CK, while environmentally adapted genera such as Sphingomonas, Corynebacterium, Acetobacter, and Lactobacillus showed elevated abundance in transgenic lines. Corn.D largely showed an intermediate genus-level composition, with Enterobacterales-associated genera abundance lower than Corn.CK but higher than Corn.S, and environmentally adapted genera abundance higher than Corn.CK but lower than Corn.S (Figure 2B). Family-level heatmap analysis showed that Enterobacteriaceae was substantially depleted in transgenic lines, with enrichment of Rhodobacteraceae and Sphingomonadaceae (Supplementary Figure S4C). At the species level, the same contrast held (Supplementary Figure S4D): the depletion was concentrated in Pantoea and Serratia. The enrichment signal, by contrast, was distributed across Sphingomonas, Corynebacterium, Acetobacter and Lactobacillus rather than concentrated in a single replacement taxon, consistent with the increase in community evenness described above. We note that some of these genera, most notably Sphingomonas, are common environmental organisms occasionally reported among low-biomass contaminants. Given that negative-control sequencing data were not available, the enrichment of these taxa should be interpreted with caution (see Discussion). Bray–Curtis cluster analysis showed a pattern of separation: Corn.CK formed a distinct cluster separate from both transgenic lines, which showed closer affinity to each other, with Corn.D clustering between Corn.CK and Corn.S (Figure 2C). The accompanying stacked bar plot showed that the wild-type phyllosphere harbored a higher proportion of Enterobacterales genera, whereas transgenic lines exhibited more diverse genus composition with increased non-Enterobacterales taxa.

Leveraging this species-level resolution, we found that the Enterobacterales depletion was driven by a specific, ecologically coherent subset of taxa rather than a uniform contraction of the order. The depleted fraction was dominated by plant- and phyllosphere-associated species, principally Pantoea (led by *P. dispersa*, which alone accounted for approximately 29% of the wild-type Enterobacterales signal, together with the maize foliar pathogen *P. ananatis* and *P. agglomerans*) and Serratia (*S. marcescens* and *S. ureilytica*), supplemented by the phytopathogens Erwinia, Dickeya and Pectobacterium. These plant-associated species declined sharply in transgenic lines and followed the same Corn.CK > Corn.D > Corn.S gradient observed at higher taxonomic ranks (Supplementary Figure S5 and Supplementary Table S1). This suggests that the Enterobacterales depletion, and the corresponding reduction in pathogenicity-associated genes described below, reflects a loss of plant-associated, partly phytopathogenic species, with Corn.D again occupying an intermediate position within this depleting clade, consistent with the intermediate taxonomic phenotype observed at phylum, order, family, and genus levels.

Together, these taxonomic analyses indicate a bidirectional remodeling pattern: depletion of Enterobacterales-associated Pseudomonadota coupled with enrichment of environmentally adapted taxa, although the latter component involves taxa that are also common environmental organisms and should be interpreted with caution in this low-biomass system. Across multiple taxonomic resolutions, Corn.D consistently occupied an intermediate position between Corn.CK and Corn.S, with the abundance of Enterobacterales-associated taxa following the gradient Corn. CK > Corn.D > Corn.S, and environmentally adapted taxa following the reverse gradient. This intermediate taxonomic profile suggests that trait stacking was associated with a microbiome composition that did not further deviate from the wild-type baseline beyond the level observed in the single-trait line.

### Pervasive metabolic reprogramming in the transgenic phyllosphere with intermediate functional profile in the stacked-trait line

Multi-level functional profiling revealed distinct metabolic reprogramming in the transgenic phyllosphere (Figure 3A). KEGG Level 2 analysis showed that most of the metabolic pathways (e.g., carbohydrate and amino acid metabolism) were enriched in Corn.CK relative to both transgenic lines. Infectious disease pathways (bacterial and viral) were also largely higher in the wild type, consistent with Enterobacterales enrichment. Notably, Corn.D exhibited an intermediate functional profile between Corn.CK and Corn.S (Figure 3A). At the KO pathway level, total classified KO abundance followed the gradient Corn.CK > Corn.D > Corn.S (Supplementary Figure S6A), reflecting higher functional gene diversity in the Enterobacterales-rich wild type. KO pathways most strongly represented in Corn.CK included xenobiotic degradation (ko00500) and two-component signal transduction (ko02020), capabilities associated with metabolically versatile taxa that sense and respond to a fluctuating environment; both were reduced in the transgenic lines (Supplementary Figure S6B). Corn.D and Corn.S clustered together in the dendrogram, with Corn.D showing a gradient between Corn.CK and Corn.S, consistent with functional congruence between the two transgenic lines and suggesting that the functional depletion is pervasive across multiple specific pathways.

**Figure 3:**
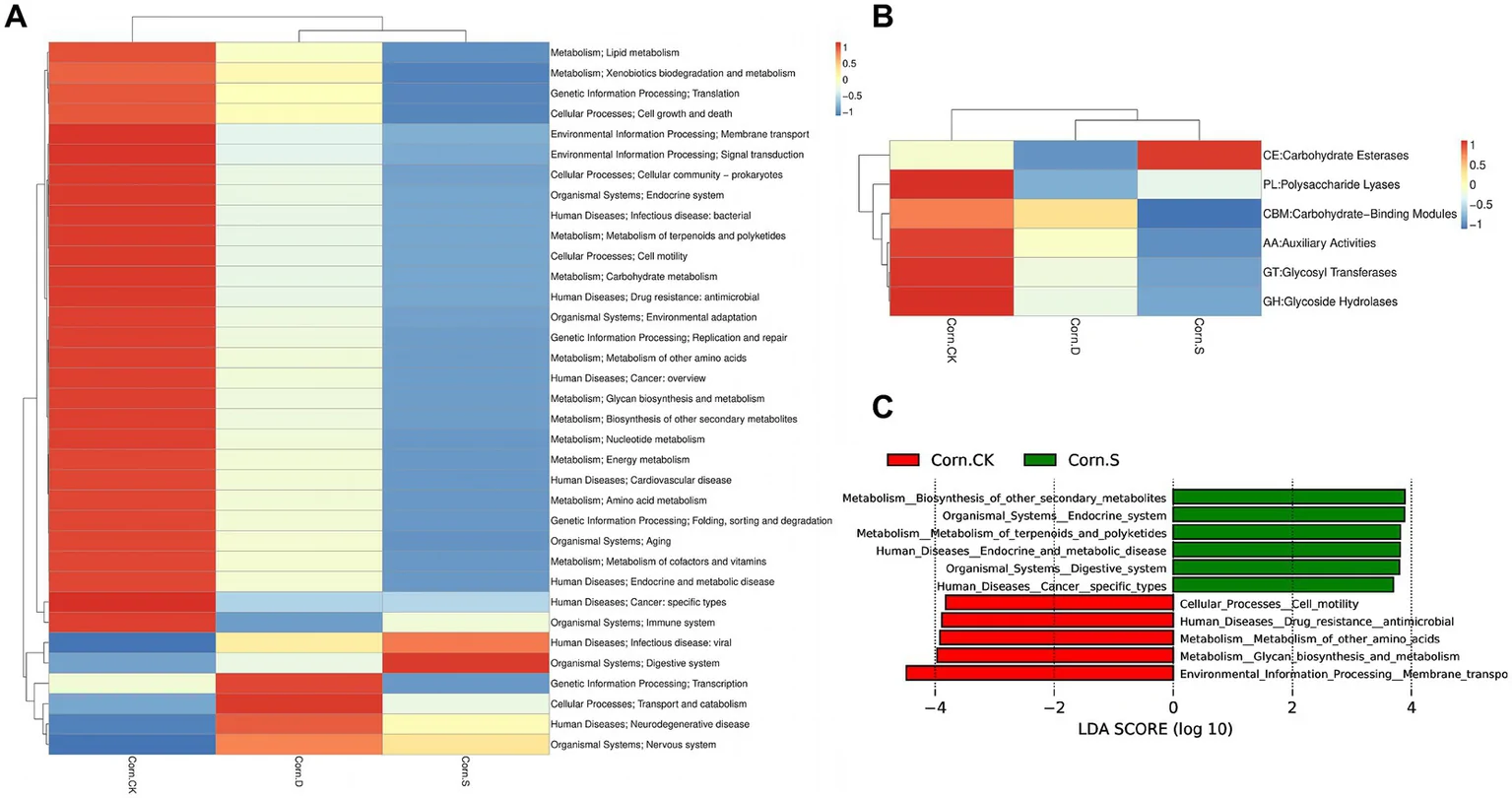
Functional profiling and LEfSe analysis of the phyllosphere microbiome across transgenic maize lines. **(A)** Heatmap showing the relative abundance of metabolic and functional pathways across Corn.CK, Corn.S, and Corn.D groups. **(B)** Heatmap displaying the relative abundance of various enzyme classes (e.g., carbohydrate esterases and glycoside hydrolases) among the three groups. **(C)** LEfSe analysis (LDA score) comparing the significantly different functional pathways between the transgenic group (Corn.S) and wild-type control (Corn.CK). Panels **(A, B)** are descriptive heatmaps of relative abundance and are not associated with significance testing. In **(C)**, LEfSe was performed with a linear discriminant analysis (LDA) score threshold of 2.0 and a significance level of *p* < 0.05 within the LEfSe procedure (Kruskal–Wallis rank-sum test followed by LDA); no additional correction for multiple testing was applied. The LDA score is itself a measure of effect size, quantifying the magnitude of differential abundance. *n* = 3 biological replicates per group.

CAZyme category-level analysis further resolved the metabolic differentiation (Figure 3B). Among the six major CAZyme classes, including glycosyltransferases (GT), auxiliary activities (AA), glycoside hydrolases (GH), carbohydrate-binding modules (CBM), carbohydrate esterases (CE), and polysaccharide lyases (PL), five (PL, CBM, AA, GT, and GH) showed lower abundance in transgenic lines (Corn.D and Corn.S) relative to Corn.CK, while CE exhibited a distinct pattern: Corn.S showed the highest abundance, followed by Corn.CK, and Corn.D the lowest (Figure 3B). At the EC level, total CAZyme EC abundance followed the same gradient (Corn.CK > Corn.D > Corn.S; Supplementary Figure S6C). The enzyme classes most depleted in the transgenic lines were those acting on cell wall and other macromolecular substrates (e.g., EC 3.4.24.-, EC 3.1.-.-; Supplementary Figure S6D), consistent with a shift away from the degradative strategy characteristic of Enterobacterales. LEfSe analysis identified KEGG Level 2 categories significantly discriminative between groups (LDA score > 2.0; Figure 3C). Corn.CK was enriched in cell motility, drug resistance (antimicrobial), glycan biosynthesis, and membrane transport, categories consistent with Enterobacterales-dominated communities where motility and membrane transport facilitate epiphytic colonization and antimicrobial resistance supports competitive advantage. Apart from the CE class noted above, Corn.D exhibited an intermediate functional profile between Corn.CK and Corn.S across most KEGG and CAZyme categories, paralleling the intermediate taxonomic position described above and suggesting that trait stacking did not further amplify the functional depletion observed in the single-trait line.

### Reduced abundance of virulence-associated and pathogenicity-associated gene homologs in the transgenic phyllosphere

Virulence factor analysis using the VFDB database quantified the abundance of virulence-associated gene homologs in the three communities (Figure 4A). Corn.CK exhibited the highest total abundance of virulence-associated gene homologs, with major categories including LOS, flagella, capsule, and T6SS. Both transgenic lines showed reduced proportions across the majority of categories. Corn.D exhibited an intermediate composition between Corn.CK and Corn.S, while Corn.S and Corn.D displayed comparable composition profiles with no category-level differences between them. The same Corn.CK > Corn.D > Corn.S gradient held across all 14 virulence functional categories (Supplementary Figure S7), suggesting that the reduction in homolog abundance is not restricted to particular categories but extends across the full functional spectrum. These VFDB-based measurements are gene-level measures of genetic potential and do not demonstrate changes in realized virulence or pathogenicity.

**Figure 4:**
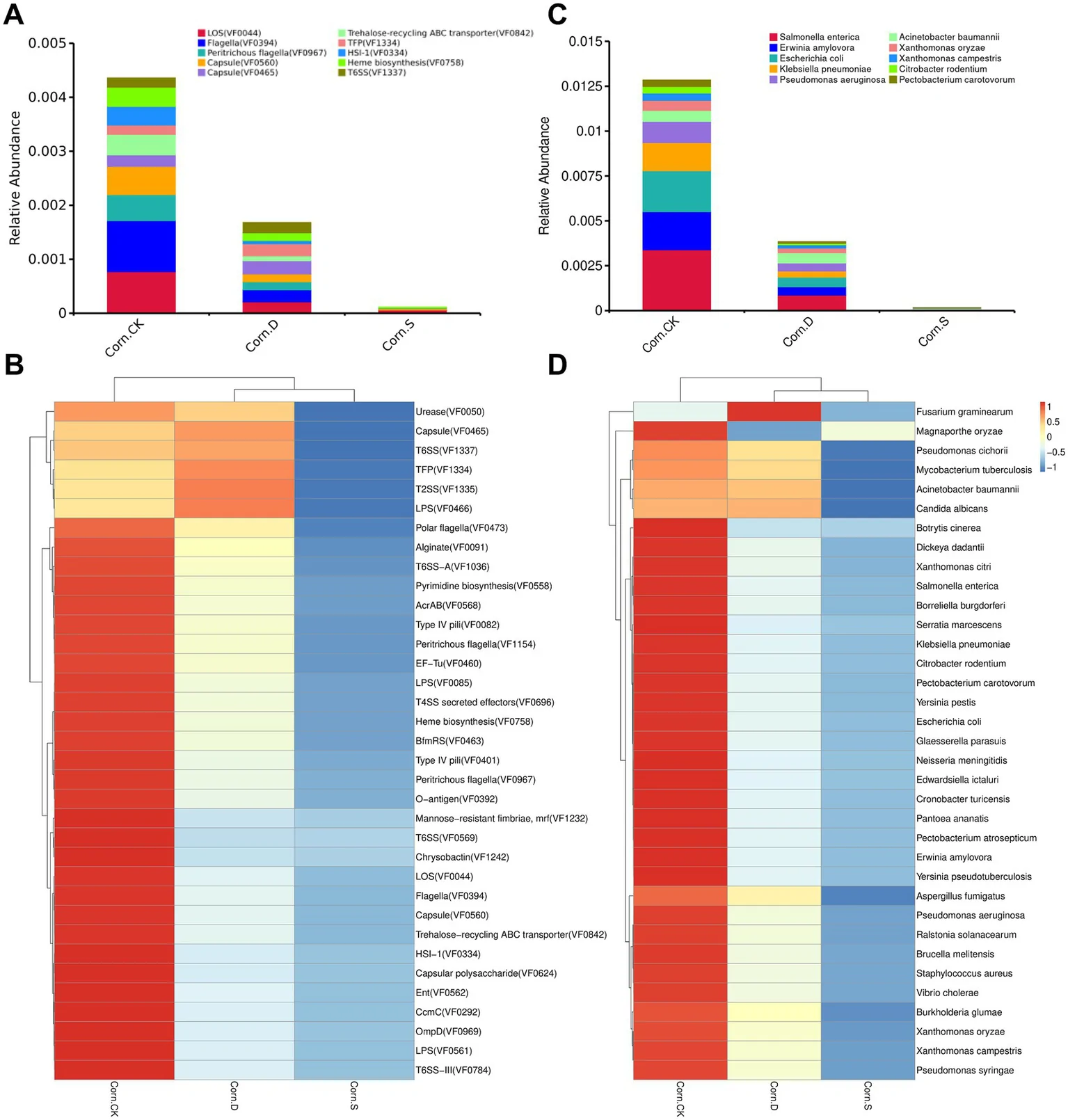
Virulence-associated and pathogenicity-associated gene homolog analysis of the phyllosphere microbiome across transgenic maize lines. **(A)** Relative abundance of VFDB-annotated virulence-associated gene homologs (stacked bar chart) showing the distribution of major virulence functional categories, including LOS, Flagella, Capsule, T6SS, and others across groups. **(B)** Heatmap with hierarchical clustering showing abundance patterns of virulence-associated genes across groups. **(C)** Relative abundance of unigenes homologous to pathogenicity-associated genes characterized in the named reference organisms (PHI-based gene-level assignments; stacked bar chart). **(D)** Heatmap with hierarchical clustering showing the abundance profiles of pathogenicity-associated gene homologs across groups. All panels are descriptive summaries of homolog abundance and are not associated with significance testing. *n* = 3 biological replicates per group.

Gene-level heatmap analysis provided finer resolution of the functional differentiation (Figure 4B). The majority of virulence-associated genes, including those encoding T6SS variants, Enterobactin (Ent), Chrysobactin, LOS, flagella, capsule, and LPS-associated factors, showed higher abundance in Corn.CK. The higher abundance of Enterobactin and multiple T6SS homologs in the wild type is noteworthy. Enterobactin is known to mediate iron acquisition in the iron-limited phyllosphere, and T6SS mediates inter-microbial competition, functions characteristic of a competitive, epiphytic lifestyle. Some specific genes such as LPS (VF0466), T2SS (VF1335), and TFP (VF1334) exhibited a distinct gradient: highest in Corn.D, intermediate in Corn.CK, and lowest in Corn.S. This pattern highlights Corn.D’s distinct gene-level profile, retaining partial abundance of virulence-associated homologs relative to both the wild type and the single-trait line. These genes, involved in membrane integrity and protein secretion, may reflect the intermediate taxonomic position of Corn.D rather than enhanced pathogenicity, as their abundance remains within the range spanned by Corn.CK and Corn.S. Furthermore, transgenic lines showed reduced abundance of stress-tolerance factors, including the trehalose-recycling ABC transporter (VF0842) and capsular polysaccharide-related factors (VF0624), which are involved in osmotic stress resistance and cell membrane integrity. Hierarchical clustering grouped Corn.S and Corn.D, separate from Corn.CK (Figure 4B).

Annotation against the pathogen–host interaction (PHI) database resolved the pathogenicity-associated gene content of the three communities (Figure 4C). These are gene-level assignments: each label denotes unigenes homologous to pathogenicity-associated genes characterized in the named reference organism, and not the detection of that organism itself. Corn.CK carried the highest abundance of such genes, most of them homologous to genes characterized in Enterobacterales reference pathogens (*Salmonella enterica*, *Escherichia coli*, *Erwinia amylovora*, and *Klebsiella pneumoniae*). Both transgenic lines carried substantially less, with Corn.D intermediate and Corn.S the lowest of the three (Figure 4C). Gene-level heatmap analysis showed the same pattern for homologues of *Dickeya dadantii*, *Yersinia pestis*, *K. pneumoniae*, *E. coli*, *E. amylovora,* and *Pantoea ananatis* genes (Figure 4D), consistent with the enrichment of Enterobacterales-associated virulence factors (Enterobactin, T6SS) in the wild type. Because pathogenicity-associated genes are widely shared across Enterobacterales, this decline is consistent with the loss of the Enterobacterales populations documented above rather than with a change in the abundance of these reference organisms themselves.

### Reduced antibiotic resistance gene diversity in transgenic maize phyllosphere with an intermediate profile in the stacked-trait line

To comprehensively evaluate the biosafety implications of transgenic events on the phyllosphere microbiome, we profiled the antibiotic resistome using the Comprehensive Antibiotic Resistance Database (CARD). ARG relative percentage analysis revealed the compositional distribution of antibiotic resistance genes across the three maize lines (Figure 5A). Notably, Corn. S exhibited a markedly reduced ARG diversity, with most categories present at low relative abundance, whereas Corn.D and Corn.CK retained comparatively more diverse ARG profiles, a pattern broadly consistent with the concomitant loss of Enterobacterales hosts, though other ecological drivers cannot be excluded. Corn.CK showed a higher relative proportion of adeF, AAC(6)-Ic, tet(41), FosA8, and qacG compared to Corn.D, consistent with the enrichment of Enterobacterales-associated taxa in the wild-type phyllosphere, as many Enterobacterales species harbor these resistance determinants. In contrast, Corn.D showed relatively higher proportions of rsmA and *Acinetobacter baumannii* AmvA, which are associated with broad-spectrum antibiotic efflux and environmental stress tolerance.

**Figure 5:**
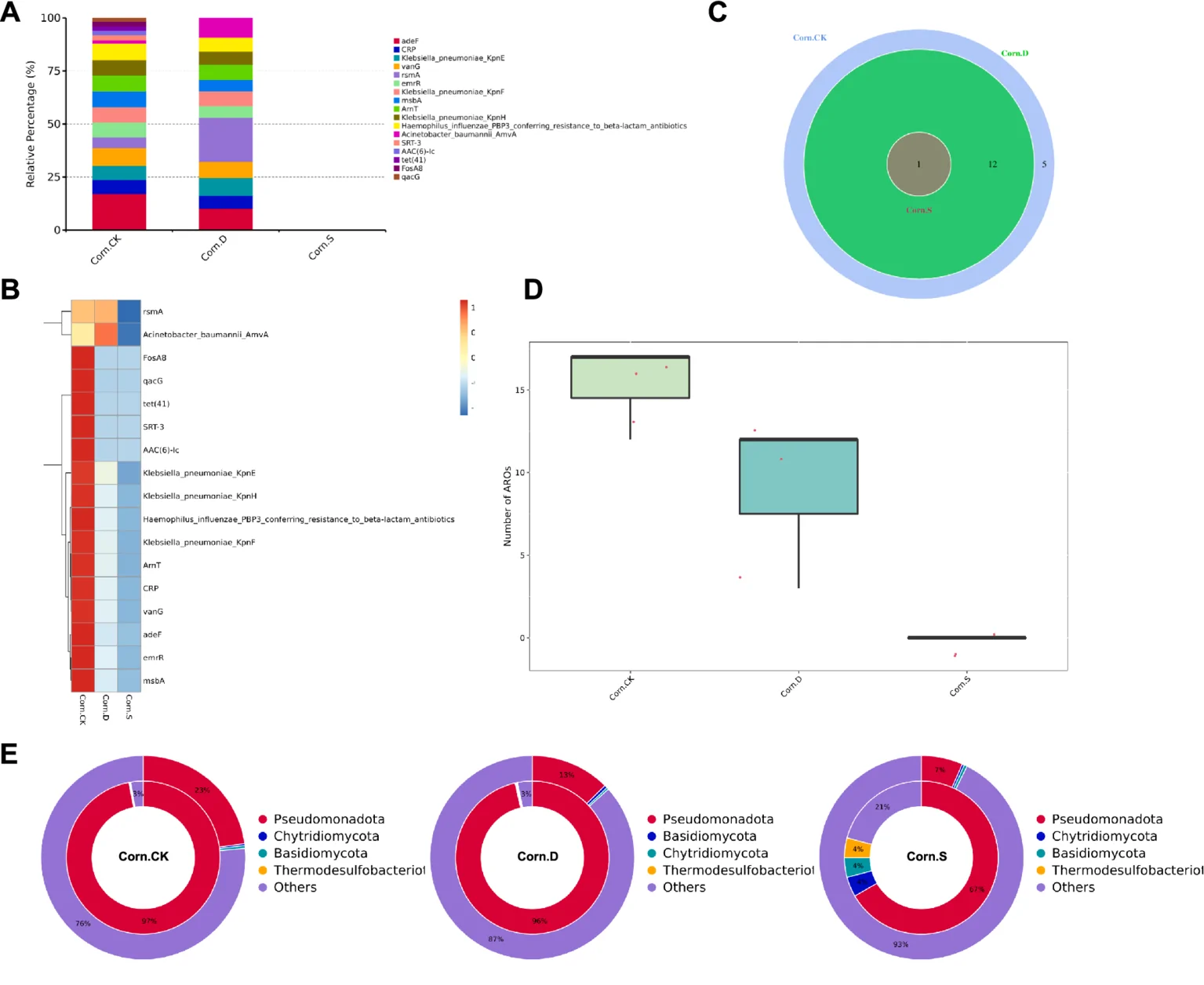
Antibiotic resistance gene (ARG) analysis of the phyllosphere microbiome across transgenic maize lines. **(A)** ARG relative percentage (stacked bar chart) showing the composition of antibiotic resistance genes in Corn.CK, Corn.D, and Corn.S groups. **(B)** ARG abundance heatmap with hierarchical clustering revealing resistance gene distribution patterns. **(C)** Venn diagram illustrating the shared and unique Antibiotic Resistance Ontology (ARO) categories among groups. **(D)** Box plot comparing the number of AROs across groups. **(E)** Circular plots showing the relative abundance of microbial phyla (e.g., *Pseudomonadota*, *Chytridiomycota*, *Basidiomycota*, *Thermodesulfobacteriota*, and Others) in each group. All panels are descriptive summaries of ARG composition and diversity and are not associated with significance testing; the box plot in **(D)** shows the distribution of ARO counts across the three biological replicates of each group. *n* = 3 biological replicates per group.

ARG gene-level heatmap analysis with hierarchical clustering provided finer resolution of the resistome differentiation between the wild-type and transgenic phyllosphere communities (Figure 5B). The heatmap revealed that the majority of resistance genes exhibited lower relative abundance in the transgenic lines Corn.D and Corn.S (versus Corn.CK). Specifically, vancomycin resistance genes (vanG, vanA) and the erythromycin resistance regulator (ermR) displayed higher relative abundance in Corn.CK, while the antibiotic resistance-associated gene (rsmA) and multidrug efflux pump gene (*Acinetobacter baumannii* AmvA) maintained high abundance in Corn.D (Figure 5B). This is consistent with the compositional analysis (Figure 5A). Hierarchical clustering of ARG abundance profiles grouped Corn.S and Corn.D together, separate from Corn.CK, indicating that the two transgenic lines share more similar resistome profiles than either does with the wild type control.

Venn diagram analysis of Antibiotic Resistance Ontology (ARO) categories revealed the shared and unique resistome components across the three groups (Figure 5C). Only one ARO category was shared among all three groups, indicating a limited core resistome across the phyllosphere communities. Corn.CK possessed the highest number of unique ARO categories, reflecting a more diverse resistome in the wild-type phyllosphere, which is consistent with the higher microbial diversity and the enrichment of Enterobacterales-associated pathogens harboring diverse resistance determinants. Corn.D exhibited a moderate number of unique AROs, while Corn.S showed the fewest unique categories. The limited pairwise overlap between groups further underscores the distinctiveness of the resistome composition across genotypes. Box plot comparison of the total number of ARO categories per sample was consistent with this pattern (Figure 5D). Corn.CK samples displayed the highest median number of ARO categories, followed by Corn.D, with Corn.S showing the lowest. This gradient suggests that the wild-type phyllosphere harbors a more diverse antibiotic resistance gene repertoire, while transgenic events are associated with a reduction in resistome diversity.

To contextualize the resistome data within the broader taxonomic framework, circular plots depicting the phylum-level composition of ARG-carrying microorganisms were generated for each group (Figure 5E). Pseudomonadota was the dominant phylum associated with antibiotic resistance genes across all three groups, consistent with its overall dominance in the phyllosphere microbiome. However, the relative contribution of Pseudomonadota to the resistome varied across groups: Corn.CK showed the highest proportion, followed by Corn.D, with Corn.S exhibiting a comparatively lower Pseudomonadota contribution. Minor phyla, including Chytridiomycota, Basidiomycota, and Thermodesulfobacteriota, contributed to the resistome in varying proportions across groups, reflecting the taxonomic remodeling patterns observed in the community-level analysis. Overall, the resistome composition of Corn.D exhibited an intermediate profile between Corn.CK and Corn.S, suggesting that the stacked-trait line showed an ARG-carrying microbial phylum composition that did not further deviate from the wild-type baseline beyond the level observed in the single-trait line. This intermediate resistome profile is consistent with the intermediate patterns observed in most of the taxonomic, functional, and virulence analyses described above.

### Mobile genetic elements and their co-occurrence with ARGs

Because the dissemination potential of a resistome is generally associated with ARGs being in proximity to mobile genetic elements (MGEs), we profiled integrons (INTEGRALL), insertion sequences (ISfinder), and plasmids and examined their co-occurrence with the CARD-based resistome. All three MGE classes followed the same genotype gradient as the ARGs, with the highest abundance in the wild type, an intermediate level in the stacked-trait line, and near-complete loss in the single-trait line (integrons, insertion sequences and plasmids: Corn.CK > Corn.D > Corn.S; Supplementary Figure S8A and Supplementary Table S2). Relative to Corn.CK, the total MGE pool was reduced by approximately 75% in Corn.D and by approximately 100% in Corn.S. ARG and MGE abundances were strongly and positively correlated across samples (Spearman’s *r* = 0.98 for the total MGE pool, and *r* = 0.98, 0.98 and 0.97 for integrons, insertion sequences and plasmids, respectively; *p* < 0.001; Supplementary Figure S8B), and 12 of the 17 detected ARGs co-occurred significantly with at least one MGE class (*r* > 0.6, FDR < 0.05; Supplementary Figure S8C). The dominant MGE carriers were themselves Enterobacterales: the most abundant integrons, insertion sequences, and plasmids were annotated to *Serratia*, *Pantoea*, *Klebsiella*, *Enterobacter*, and *Salmonella*. This covariation, together with the Enterobacterales affiliation of the dominant MGE annotations, is consistent with the ARGs being preferentially associated with Enterobacterales lineages that also carry MGEs, although the present co-occurrence data do not demonstrate physical linkage between ARGs and MGEs. In terms of abundance, the depletion of these hosts in transgenic lines was accompanied by coordinated reductions in both ARGs and MGEs. At the level of co-occurrence, the addition of the MGE dimension is consistent with, rather than contradicting, the resistome findings: the abundance of ARGs co-varying with MGEs, and with it the putative potential for horizontal ARG dissemination, is reduced (intermediate in Corn.D, near-absent in Corn.S) rather than amplified by trait stacking, pending confirmation by direct linkage evidence.

## Discussion

This study characterizes the phyllosphere microbiome of a stacked-trait maize line (Corn.D, *cry1Ab* + *cp4-epsps*) relative to its wild-type (Corn.CK) and *cp4-epsps* single-trait (Corn.S) near-isogenic counterparts, and shows that the stacked-trait line exhibited an intermediate microbiome profile between the other two lines across several of the analytical dimensions examined, which we interpret as a consistent descriptive trend given the sample size (Figure 6). By employing herbicide-free conditions, we removed a major agrochemical confounder, an important methodological advance, as herbicide application often masks or synergizes with transgenic effects on plant microbiomes (Newman et al., 2016). Our findings raise the possibility that, for this genotype combination, the stacked-trait line does not lie further from the wild-type baseline than the single-trait line does, an observation that now warrants testing under field conditions.

**Figure 6:**
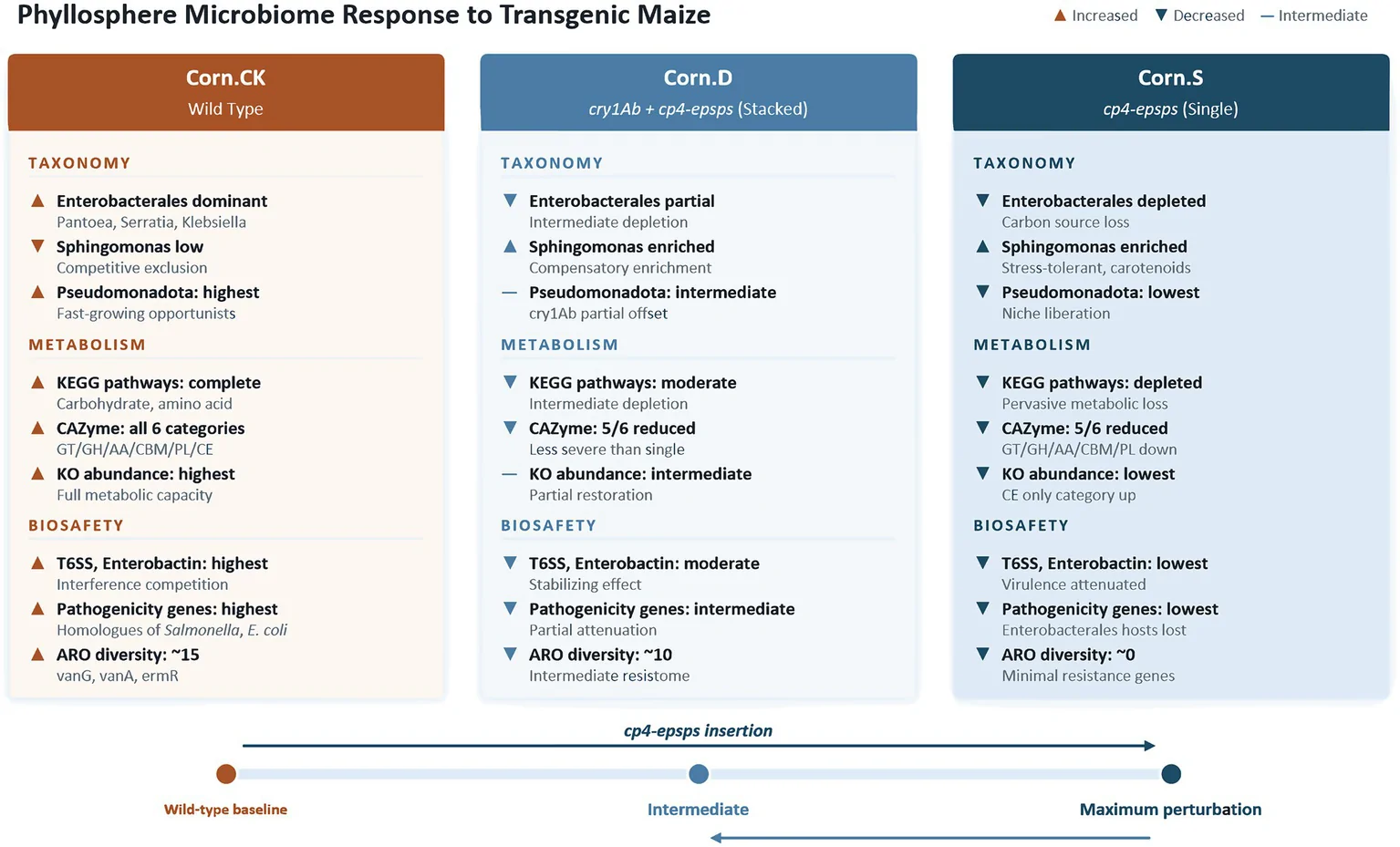
Graphical summary of the observed phyllosphere microbiome patterns across the three maize lines. The three genotypes are arranged from left to right along a gradient of perturbation relative to the wild-type baseline (lower axis), rather than in the order in which the transgenes were introduced: Corn.CK (wild type) marks the baseline, Corn.S (*cp4-epsps*, single trait) the largest departure from it, and Corn.D (*cry1Ab* + *cp4-epsps*, stacked trait) an intermediate position between the two. Each panel summarizes the observations for that genotype across the analytical dimensions examined in this study—taxonomic composition, metabolic potential, and the virulence factors and antibiotic resistome grouped under Biosafety—with ▲, ▼, and—denoting, respectively, the highest, the lowest, and an intermediate value among the three lines. The two arrows graphically summarize the observed ordering of the three genotypes along this gradient: the upper arrow denotes that Corn.S lies farthest from the wild-type baseline, and the lower arrow denotes that Corn.D lies between Corn.CK and Corn.S. The arrows do not represent measured or inferred causal effects of the transgenes; positions along the gradient are schematic rather than quantitative. Interpreting the upper arrow as an effect of *cp4-epsps* insertion, or the lower arrow as a counteracting effect of *cry1Ab*, would be an untested hypothesis requiring additional comparator lines (including a *cry1Ab*-only line) and direct mechanistic data (see Discussion). All comparisons are based on *n* = 3 biological replicates per genotype, grown under herbicide-free greenhouse conditions and sampled at the V7 stage.

The Shannon-index increase observed in the *cp4-epsps* single-trait line relative to the wild type (a consistent trend with a large effect size, Cliff’s δ = +1.00; exact permutation *p* = 0.100, the minimum attainable two-sided value at *n* = 3 per group; not significant after correction for multiple comparisons), together with the intermediate but non-significant increase in the stacked-trait line, contrasts with the conventional paradigm that genetic modification reduces microbial diversity. Ecologically, this increase may reflect a release from competitive dominance. The depletion of Enterobacterales, an opportunistic and fast-growing lineage that typically dominates the niche via interference competition (e.g., T6SS) and iron scavenging (Enterobactin), would be expected to liberate niche space. This potentially allowed for the enrichment of environmentally adapted, stress-tolerant taxa, resulting in higher community evenness. The decoupling of evenness and richness (Chao1: stacked > wild > single) aligns with previous reports suggesting that ephemeral microbial communities, like the phyllosphere, are structured by stochastic assembly and priority effects (Vorholt, 2012).

We propose, as one plausible but as-yet-untested hypothesis, that the bidirectional taxonomic remodeling may be linked to alterations in leaf metabolic profiles associated with transgene expression. This mechanistic explanation is not supported by direct experimental data in the present study and would require validation through metabolomic or transcriptomic profiling of leaf exudates. One hypothesized pathway-level consequence of cp4-epsps expression is an alteration of flux through the shikimate pathway, potentially depleting aromatic amino acid-derived secondary metabolites (e.g., phenolics) that normally mediate host–microbe interactions and shape the phyllosphere epiphytic lifestyle (Newman et al., 2016). Under this hypothesis, Enterobacterales, which heavily rely on specific host-derived carbon sources, may be disadvantaged by this metabolic shift. Conversely, *Sphingomonas*, known for degrading complex organic compounds and producing carotenoids against oxidative stress (Delmotte et al., 2009), would be comparatively well placed to persist if the leaf environment were altered in this way. The intermediate taxonomic position of the stacked-trait line is consistent with the possibility that the leaf metabolic environment of Corn.D lies between those of Corn.CK and Corn.S, but the present design cannot determine whether this position arises from an interaction between the two transgenes or from the independent effects of each. As one untested hypothesis, the metabolic burden of insecticidal protein synthesis might modify the host resource landscape relative to the *cp4-epsps*-only state. Evaluating this hypothesis would require direct metabolomic or transcriptomic measurements of leaf exudates, together with a *cry1Ab*-only comparator. These mechanistic considerations are accordingly offered solely as speculative hypotheses. They are not evidence for the compensatory buffering effect discussed below, for which no host metabolomic or transcriptomic data are available in this study.

Functionally, the pervasive depletion of metabolic pathways (KEGG and CAZymes) alongside taxonomic shifts did not precipitate ecosystem collapse, underscoring the principle of functional redundancy in the phyllosphere (Louca et al., 2018). While metabolically versatile Enterobacterales were depleted, enriched taxa maintained core biogeochemical cycling, compensating through specific stress-tolerance factors (e.g., rsmA and AmvA) and carbohydrate esterases. This functional plasticity suggests that profound taxonomic turnover does not necessarily translate to proportional functional disruption.

From a biosafety perspective, the concurrent reduction in the abundance of virulence-associated gene homologs and in antibiotic resistance gene diversity associated with Enterobacterales is noteworthy. These are, however, measures of genetic potential: metagenomic sequencing quantifies the abundance of virulence-associated and resistance genes, not their expression, nor the realized pathogenicity or resistance phenotype of the organisms carrying them. Read at that level, the reduction of T6SS and Enterobacterales-associated pathogen sequences is consistent with transgenic events inadvertently reducing the genetic potential for pathogenicity carried by this lineage. This aligns with the “competition-colonization trade-off” theory: the removal of competitive Enterobacterales reduces this specific reservoir of opportunistic pathogens and their co-occurring antibiotic resistance determinants (Chen et al., 2019). However, it is crucial to acknowledge that the niche liberation caused by Enterobacterales depletion could potentially be occupied by other unknown or emerging opportunistic taxa, warranting long-term monitoring. The predominantly intermediate virulence and resistome gene profiles of the stacked-trait line parallel the intermediate patterns observed in most of the taxonomic and functional analyses described above. The elevated abundance of specific membrane integrity genes (LPS, T2SS) in the stacked line most likely reflects its intermediate taxonomic composition; as with the reductions described above, however, it is evidence neither of a novel pathogenic risk nor of its absence. Establishing whether any of these gene-level shifts translates into a change in biosafety risk would require transcriptomic or culture-based validation.

The intermediate position of Corn.D between Corn.CK and Corn.S is the central descriptive finding of this study. Relative to the wild-type baseline, the stacked-trait line did not deviate further from the wild-type than the single-trait line did, and in most analyses it occupied an intermediate position between the two. One possible, as-yet-untested interpretation of this descriptive pattern is a “compensatory buffering effect,” whereby the combined effects of the two transgenes on the host plant would differ from those of *cp4-epsps* alone, positioning the community between the wild-type and single-trait baselines. This is stated solely as a hypothesis: the host-metabolic considerations outlined above are not evidence for it, as no host metabolomic or transcriptomic data were obtained in this study, and testing it would require a complete factorial design including a *cry1Ab*-only near-isogenic line, together with direct metabolic profiling of the host. Moreover, given the constraints of the existing dataset (*n* = 3 per group), this intermediate-phenotype pattern should be regarded as a consistent trend across independent analytical dimensions, rather than as a statistically confirmed effect.

Because the phyllosphere is a low-biomass habitat, we considered whether contamination could account for the observed shifts. Raw sequence data from the negative controls could not be obtained from the sequencing provider, so contamination could not be directly quantified in this study; the assessment below therefore rests on indirect, pattern-based evidence and should be read as suggestive rather than conclusive. Within this constraint, several observations are at least not consistent with a dominant role of sample-independent background contamination. First, community structure was genotype-structured rather than random, with replicates of each genotype clustering together and the wild type separating from both transgenic lines (Figures 2C,D). Second, the genera driving our conclusions, the Enterobacterales-associated *Pantoea* and *Serratia*, do not appear on established reagent-contaminant catalogues (Salter et al., 2014; Eisenhofer et al., 2019), and their depletion followed a consistent genotype-dependent gradient. The enrichment side of the remodeling pattern requires more caution. Sphingomonas, the most prominent enriched taxon, is both a well-known phyllosphere dweller and a genus occasionally reported among low-biomass contaminants (Delmotte et al., 2009). Its enrichment in the transgenic lines was directional rather than uniform across samples, which is more consistent with a biological response than with a constant background signal. However, in the absence of sequenced negative controls, a contribution of contamination to the observed abundance of this and other low-abundance taxa cannot be excluded, and claims concerning these taxa are correspondingly tentative.

In addition, several limitations must be acknowledged. First, the small sample size (n = 3 per group) limits statistical power, as evidenced by non-significant ANOSIM results for beta diversity. This limitation applies broadly to the intermediate-phenotype claims made throughout this study, which are best interpreted as consistent trends rather than statistically confirmed effects. It also prevents us from definitively concluding a strict “zero effect” between single and stacked traits. Larger cohorts are essential to validate these preliminary patterns. Second, the absence of a *cry1Ab*-only near-isogenic line means that the individual effect of *cry1Ab* could not be estimated. Consequently, the intermediate phenotype of the stacked-trait line cannot be attributed to a specific interaction between the two transgenes, and formal tests of additivity, antagonism, or synergy would require a complete factorial genetic design. Third, the mechanistic links between transgene expression and microbial remodeling remain hypothesized. No host metabolomic or transcriptomic data were collected, and direct evidence through metabolomic profiling of leaf exudates is required. Fourth, the temporal dynamics of the observed patterns were not assessed. Phyllosphere communities undergo successional changes with leaf age, and the perturbation intensity of transgenic traits may vary across developmental stages. Fifth, the herbicide-free greenhouse design, which minimizes agrochemical confounding, also limits the transferability of the result: a phyllosphere without glyphosate selection pressure or insect herbivory is ecologically distinct from a field canopy. More broadly, the microbiome profile reported here derives from one genotype combination, one developmental stage (V7), one controlled environment, and three replicates per line, and therefore cannot establish that stacked traits generally present lower or equivalent microbial risk. The two transgenes may also interact differently in other genetic backgrounds, and the pattern reported here should accordingly be regarded as a hypothesis for field testing rather than as a general property of stacked-trait crops. Finally, raw sequence data from the negative controls could not be obtained from the sequencing provider. Consequently, contamination could not be directly quantified, and the contamination assessment relies on indirect, pattern-based inference. This limitation is most relevant to low-abundance taxa, in particular Sphingomonas and other genera occasionally reported among low-biomass contaminants, and conclusions concerning these taxa should be regarded as tentative pending validation with sequenced negative controls.

In conclusion, transgenic maize reshapes the phyllosphere via bidirectional taxonomic remodeling and reduced abundance of functional gene categories, including virulence-associated and resistance gene homologs. For this genotype combination, the stacked-trait line exhibited a predominantly intermediate microbiome profile between the wild-type and cp4-epsps single-trait lines, suggesting that trait stacking did not exacerbate microbial perturbation beyond that caused by the single trait alone. By removing a major agrochemical confounder under herbicide-free greenhouse conditions, this study provides a preliminary, genotype-specific reference for the phyllosphere microbiome that may inform future biosafety assessment of stacked-trait crops. Whether this intermediate phenotype reflects a specific interaction between the two transgenes, and whether it holds under field conditions, remains to be established. Field validation is therefore required before these results can inform biosafety assessment of stacked-trait crops.

## Data Availability

The datasets presented in the study are deposited in the NCBI Sequence Read Archive (SRA) repository, accession number PRJNA1475914.
