# Supplementary material for: Effects of trait stacking on the maize phyllosphere microbiome: a shotgun metagenomic comparison
Source: Front Microbiol. 2026 Sep 28;17:1904641. doi: 10.3389/fmicb.2026.1904641 (PMC13655118; doi:10.3389/fmicb.2026.1904641)
Supplement: Supplementary file 1 [file Image_1.pdf]

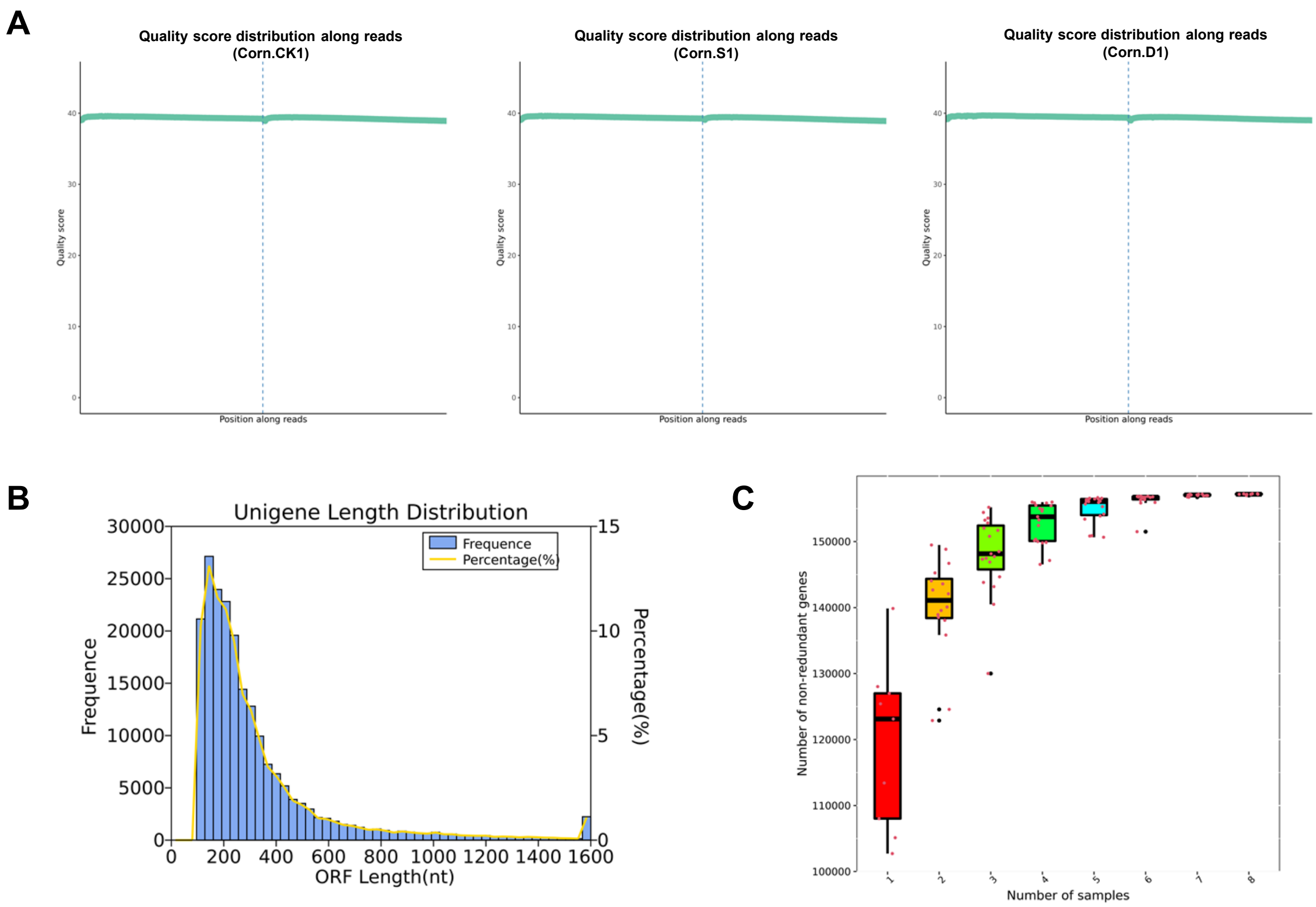

Figure S1. Sequencing quality assessment and gene catalog construction. (A) Quality score distribution along reads for representative samples from each group (one replicate each of Corn.CK, Corn.S and Corn.D), showing consistently high quality scores across the entire read length. (B) Unigene length distribution showing the frequency (bars) and percentage (line) of predicted open reading frames (ORFs) across different length intervals (nt). (C) Number of non-redundant genes identified across samples, displayed as box plots with individual data points. Panels (A)–(C) are quality-control summaries and are not associated with significance testing.

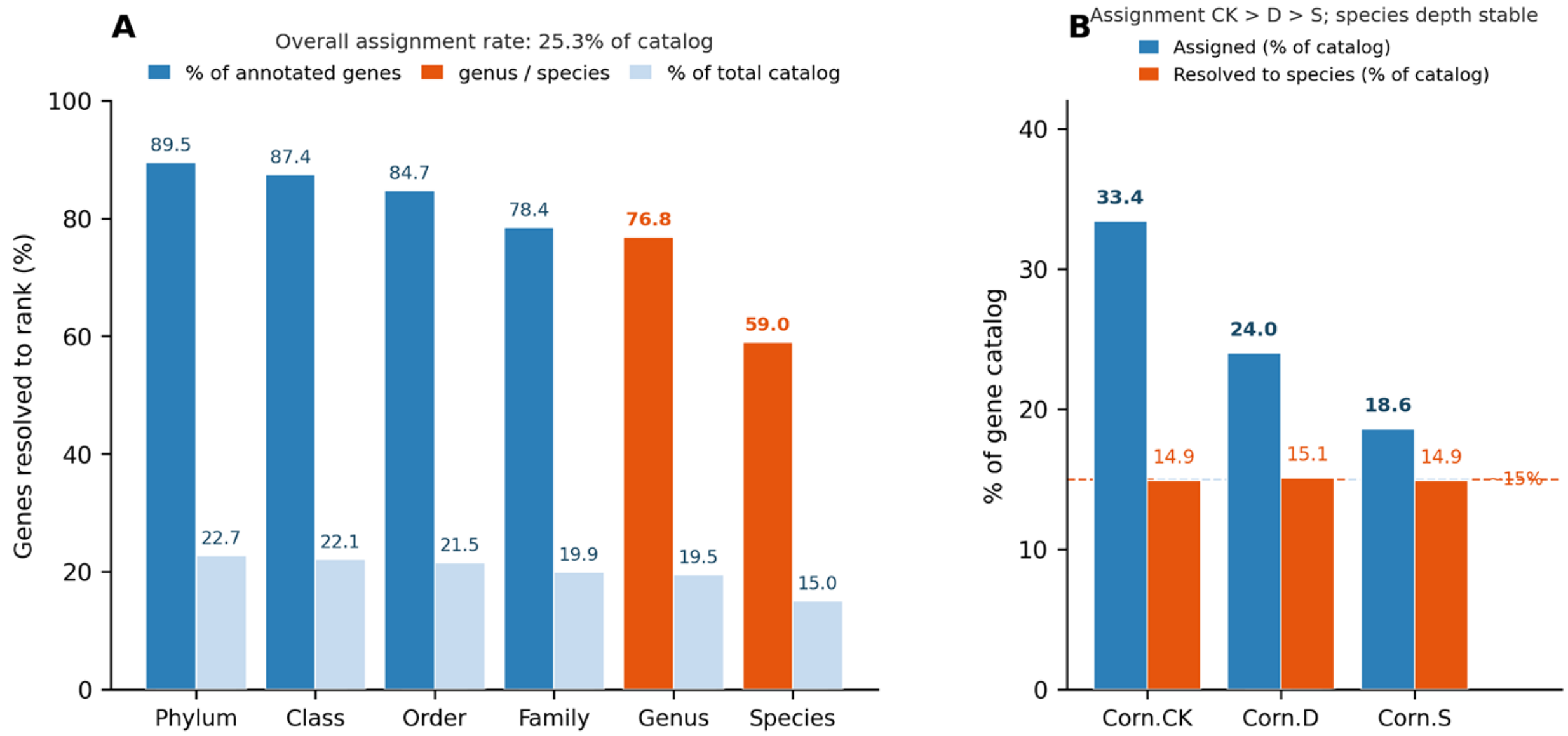

Figure S2. Taxonomic annotation resolution of the non-redundant gene catalog. (A) Percentage of genes resolved to each taxonomic rank (phylum to species), shown both as a fraction of the taxonomically annotated genes (dark bars; genus and species highlighted) and as a fraction of the entire gene catalog (light bars); the overall taxonomic assignment rate (25.3% of the catalog) is indicated above the panel. (B) Per-group annotation depth: the overall taxonomic assignment rate (fraction of the catalog assigned to at least the domain level) and the fraction of the catalog resolved to species, for Corn.CK, Corn.D and Corn.S (n = 3 biological replicates per line). Resolution was computed from the MEGAN lowest-common-ancestor assignments as the proportion of gene abundance classified at each rank; the ~15% species-level fraction was comparable across lines.

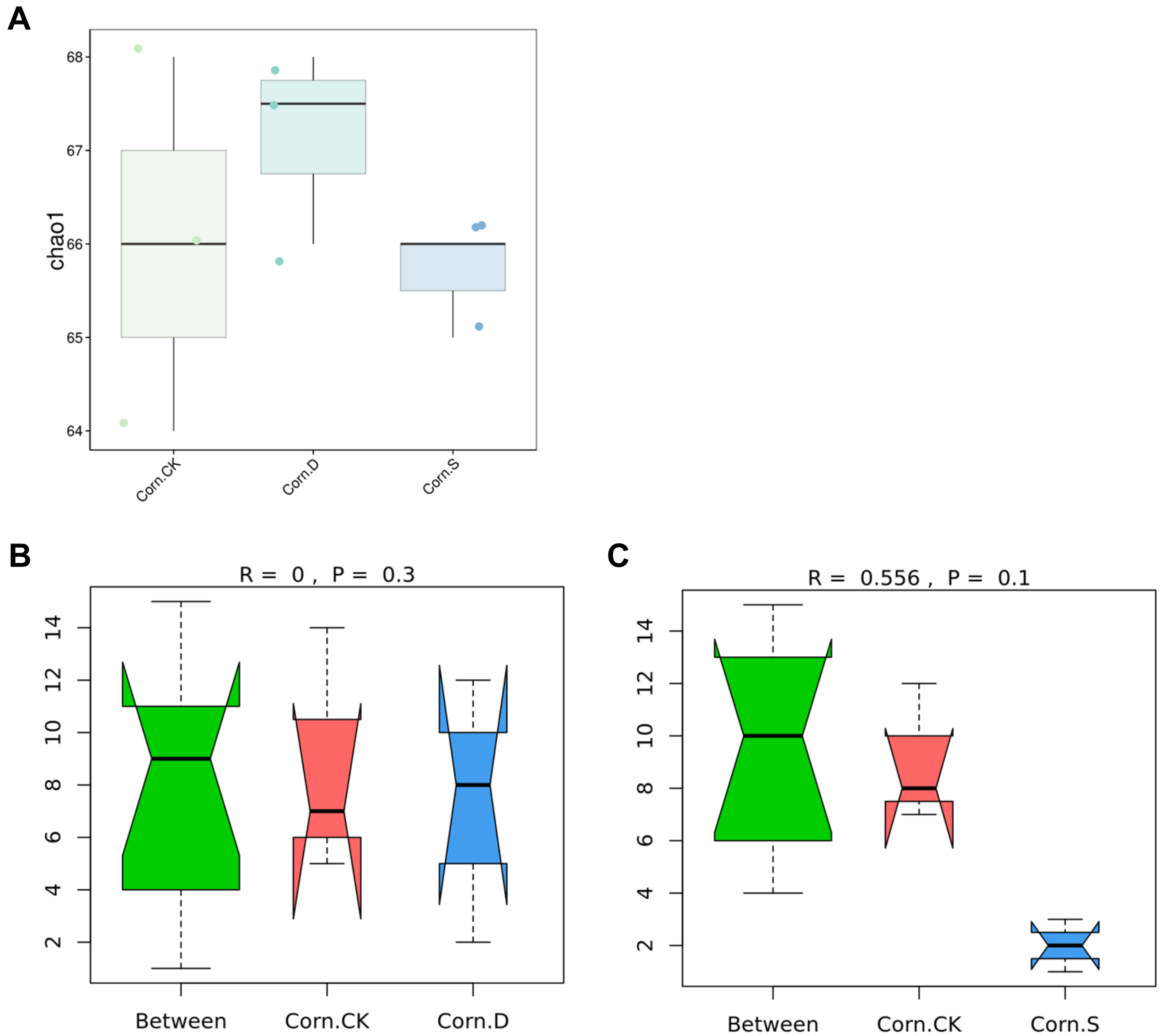

Figure S3. Supplementary diversity analysis of the phyllosphere microbiome. (A) Chao1 diversity index comparison among Corn.CK, Corn.S, and Corn.D groups. Cliff's δ values (δ; positive values indicate higher values in the first-named group, tied pairs excluded) were: Corn.S versus Corn.CK, δ = −0.14 (negligible); Corn.D versus Corn.CK, δ = +0.43 (medium); Corn.D versus Corn.S, δ = +1.00 (large). (B) ANOSIM (Analysis of Similarities) results for the comparison between Corn.CK and Corn.D groups. (C) ANOSIM results for the comparison between Corn.CK and Corn.S groups (R = 0.556, P = 0.1). The Chao1 comparison in (A) was performed using the Kruskal–Wallis rank-sum test (omnibus P = 0.358; exact permutation P = 0.457, based on exhaustive enumeration of all 1,680 possible assignments of the nine samples to the three genotype groups), followed by two-sided exact two-sample permutation tests for each

pairwise contrast (exhaustive enumeration of all 20 possible allocations per contrast) with Holm–Bonferroni correction; all pairwise comparisons were non-significant (exact permutation P ≥ 0.300; Holm-adjusted P ≥ 0.900). ANOSIM in (B) and (C) was based on Bray–Curtis distance with 999 permutations. n = 3 biological replicates per group.

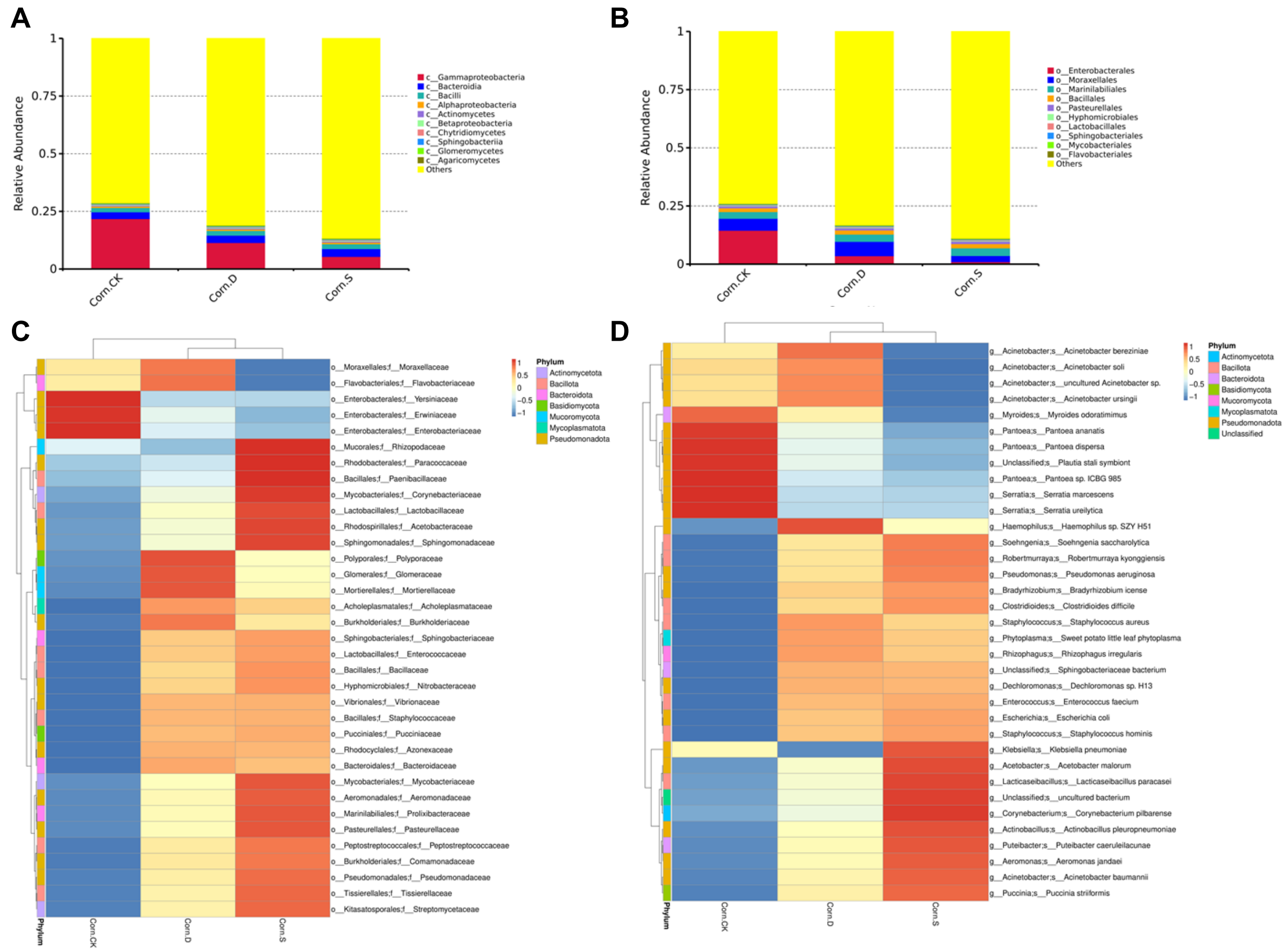

Figure S4. Supplementary taxonomic composition analysis of the phyllosphere microbiome. (A) Class-level relative abundance (stacked bar chart) across groups. (B) Order-level relative abundance (stacked bar chart) across groups. (C) Family-level heatmap with hierarchical clustering and phylum-level color annotation. (D) Species-level heatmap with hierarchical clustering and phylum-level color annotation. All panels are descriptive summaries of relative abundance and are not associated with significance testing.

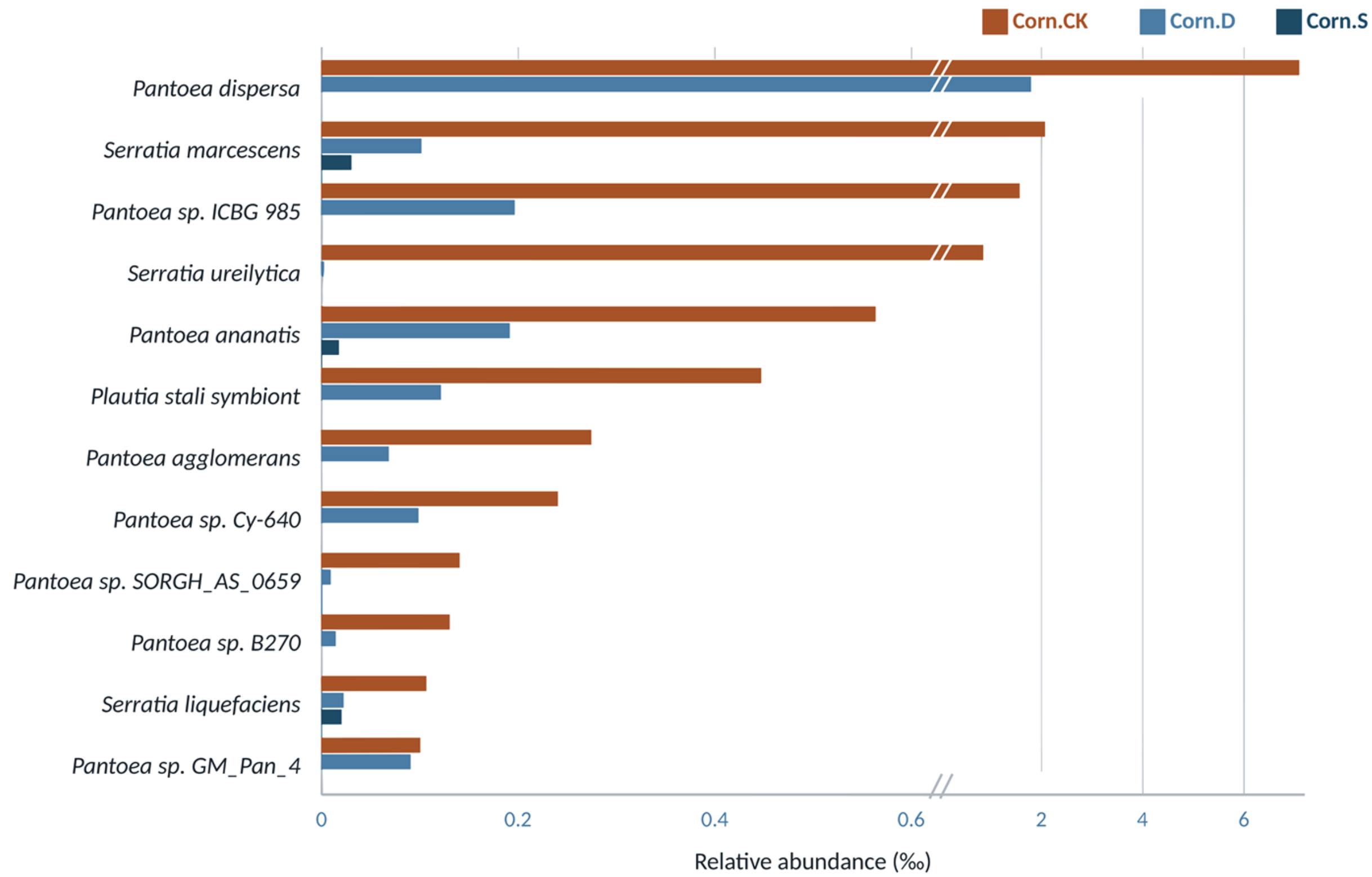

Figure S5. Species-level resolution of the Enterobacterales depletion. Relative abundance (‰ of total annotated genes) of the twelve most abundant Enterobacterales species showing a genotype-dependent response, in Corn.CK, Corn.D and Corn.S. Species are ordered by their abundance in Corn.CK. Bars are means of three biological replicates per genotype. The x-axis is broken between 0.6‰ and 2‰ (double slash) to accommodate the wide range of abundances. These twelve species account for 90% of the responsive Enterobacterales fraction, which declines from 13.56‰ in Corn.CK to 2.70‰ in Corn.D and 0.07‰ in Corn.S. The depletion is graded across the three genotypes, with Corn.D intermediate: every Corn.CK replicate exceeded every Corn.D replicate, which in turn exceeded every Corn.S replicate (Cliff's $\delta = +1.00$ for all three pairwise comparisons). Non-responsive enteric species (for example Escherichia coli and Klebsiella pneumoniae), whose abundance was unchanged across genotypes, are not shown.

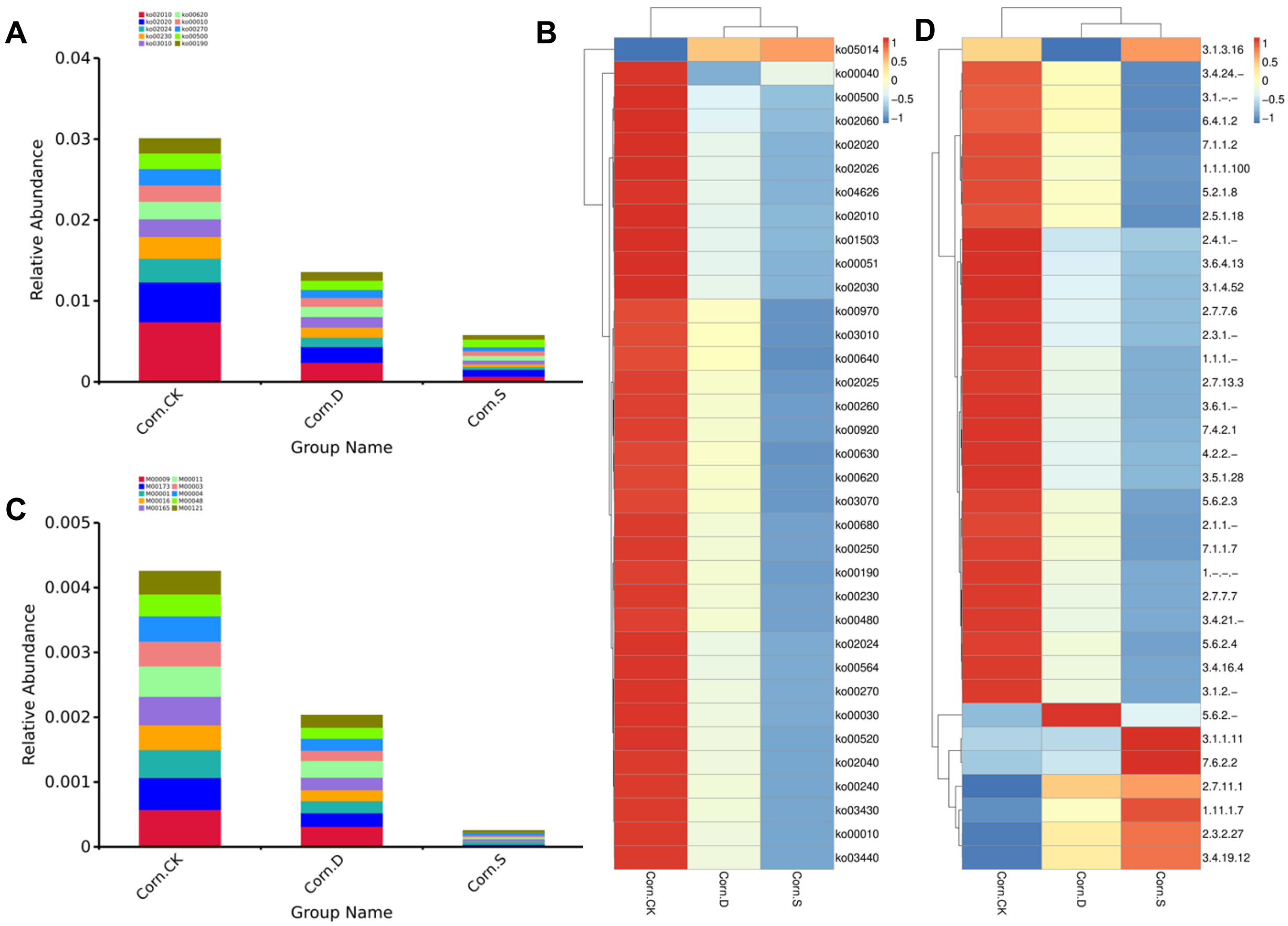

Figure S6. Supplementary functional profiling of the phyllosphere microbiome. (A) Stacked bar plot showing the relative abundance of the top 10 KEGG Level 3 pathways among Corn.CK, Corn.D, and Corn.S groups. (B) Heatmap displaying the relative abundance of KEGG Level 3 pathways across the three groups, with color intensity representing abundance levels. (C) Stacked bar plot illustrating the relative abundance of the top 10 KEGG modules among groups. (D) Heatmap presenting the relative abundance of KEGG Enzyme Commission (EC) categories across the three groups, with color indicating abundance levels. All panels are descriptive summaries of relative abundance and are not associated with significance testing.

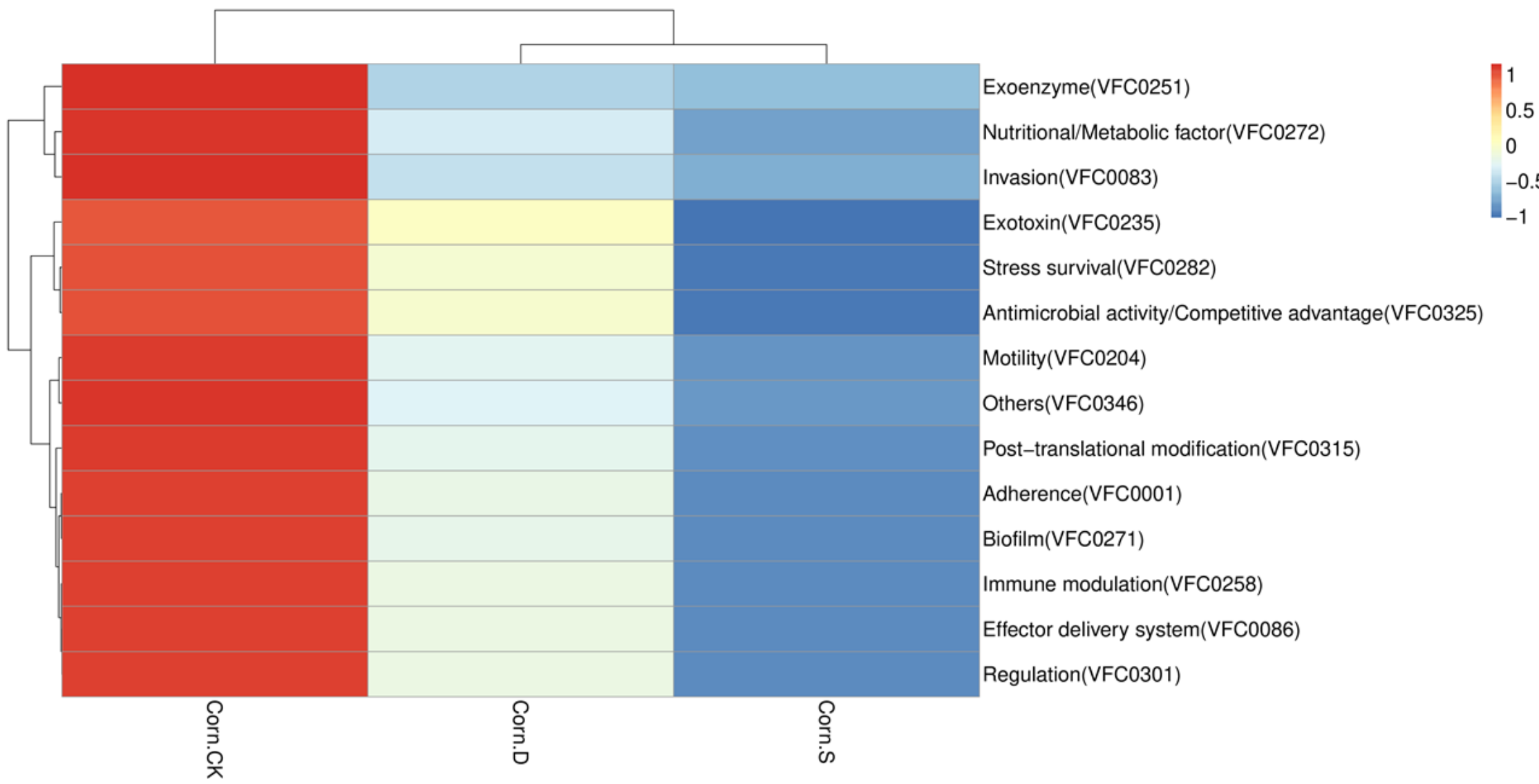

Figure S7. Supplementary virulence and antibiotic resistance analysis. VFDB virulence functional classification heatmap showing the abundance of major virulence functional categories across groups. This panel is a descriptive summary of relative abundance and is not associated with significance testing.

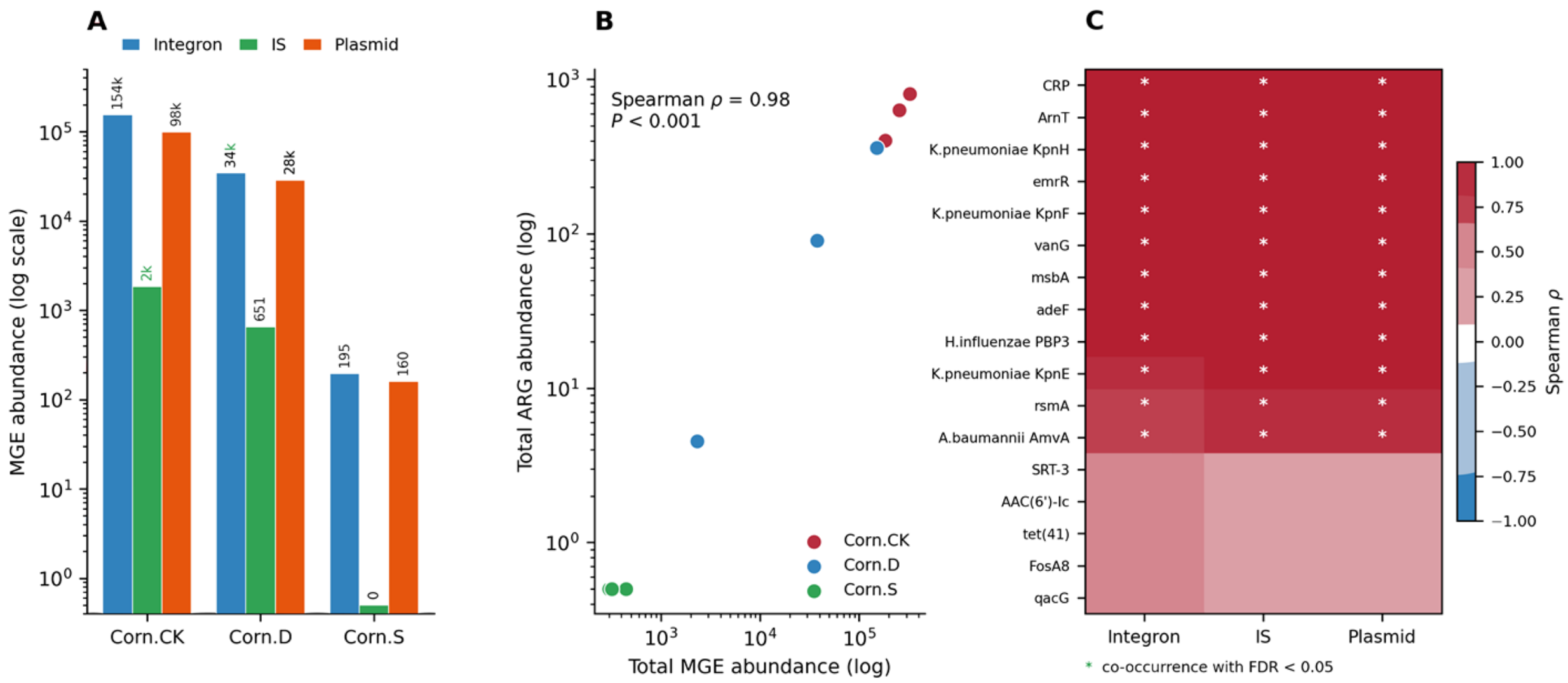

Figure S8. Co-occurrence of antibiotic resistance genes (ARGs) and mobile genetic elements (MGEs) in the phyllosphere microbiome. (A) Mean absolute abundance of the three MGE classes — integrons (INTEGRALL), insertion sequences (ISfinder) and plasmids — across the wild-type (Corn.CK), stacked-trait (Corn.D) and single-trait (Corn.S) lines (n = 3 biological replicates per line; y-axis on a log10 scale; bar labels give the group mean). (B) Co-occurrence of total ARG and total MGE abundance across all nine samples; each point is one sample, coloured by line, and the Spearman rank-correlation coefficient (ρ) is indicated. (C) Heatmap of Spearman correlations between each detected ARG and each MGE class across the nine samples; asterisks denote significant co-occurrence ($\rho \geq 0.6$ and Benjamini–Hochberg FDR < 0.05). Abundances are database-normalized gene abundances with the non-target background category excluded.
